# The experimental power of FR900359 to study Gq-regulated biological processes

**DOI:** 10.1038/ncomms10156

**Published:** 2015-12-14

**Authors:** Ramona Schrage, Anna-Lena Schmitz, Evelyn Gaffal, Suvi Annala, Stefan Kehraus, Daniela Wenzel, Katrin M. Büllesbach, Tobias Bald, Asuka Inoue, Yuji Shinjo, Ségolène Galandrin, Naveen Shridhar, Michael Hesse, Manuel Grundmann, Nicole Merten, Thomas H. Charpentier, Matthew Martz, Adrian J. Butcher, Tanja Slodczyk, Sylvain Armando, Maike Effern, Yoon Namkung, Laura Jenkins, Velten Horn, Anne Stößel, Harald Dargatz, Daniel Tietze, Diana Imhof, Céline Galés, Christel Drewke, Christa E. Müller, Michael Hölzel, Graeme Milligan, Andrew B. Tobin, Jesús Gomeza, Henrik G. Dohlman, John Sondek, T. Kendall Harden, Michel Bouvier, Stéphane A. Laporte, Junken Aoki, Bernd K. Fleischmann, Klaus Mohr, Gabriele M. König, Thomas Tüting, Evi Kostenis

**Affiliations:** 1 Molecular, Cellular and Pharmacobiology Section, Institute of Pharmaceutical Biology, University of Bonn, 53115 Bonn, Germany; 2 Pharmacology & Toxicology Section, Institute of Pharmacy, University of Bonn, 53121 Bonn, Germany; 3 Department of Dermatology and Allergy, Laboratory of Experimental Dermatology, University of Bonn, 53105 Bonn, Germany; 4 Institute of Pharmaceutical Biology, University of Bonn, 53115 Bonn, Germany; 5 Institute of Physiology I, Life and Brain Center, University of Bonn, 53105 Bonn, Germany; 6 Graduate School of Pharmaceutical Sciences, Tohoku University, 6-3, Aoba, Aramaki, Aoba-ku, Sendai City 980-0065, Japan; 7 Japan Science and Technology Agency (JST), Precursory Research for Embryonic Science and Technology (PRESTO), 4-1-8 Honcho, Kawaguchi, Saitama 332-0012, Japan; 8 Institut des Maladies Métaboliques et Cardiovasculaires, Institut Nataional de la Santé et de la Recherche Médicale, Université Toulouse III Paul Sabatier, 31432 Toulouse, France; 9 Department of Pharmacology, University of North Carolina School of Medicine, Chapel Hill, North Carolina 27599, USA; 10 Department of Biochemistry and Biophysics, University of North Carolina School of Medicine, Chapel Hill, North Carolina 27599, USA; 11 Medical Research Council Toxicology Unit, University of Leicester, Hodgkin building, Leicester LE1 9HN, UK; 12 Department of Medicine, McGill University, and the Research Institute of the McGill University Health Centre, Montreal, Quebec, Canada H4A 3J1; 13 Department of Clinical Chemistry and Clinical Pharmacology, University of Bonn, 53105 Bonn, Germany; 14 Molecular Pharmacology Group, Institute of Molecular, Cell and Systems Biology, College of Medical, Veterinary and Life Sciences, University of Glasgow, Glasgow, Scotland G12 8QQ, UK; 15 Eduard-Zintl-Institute of Inorganic and Physical Chemistry, Technische Universität Darmstadt, 64287 Darmstadt, Germany; 16 PharmaCenter Bonn, Pharmaceutical Institute, Pharmaceutical Chemistry I, University of Bonn, 53121 Bonn, Germany; 17 Pharmaceutical Chemistry I, Institute of Pharmacy, University of Bonn, 53121 Bonn, Germany; 18 Department of Biochemistry and Institute for Research in Immunology and Cancer, Université de Montréal, Montreal, Québec, Canada QC H3C IJ4; 19 Japan Agency for Medical Research and Development, Core Research for Evolutional Science and Technology (AMED-CREST), 1-7-1 Otemachi, Chiyoda, Tokyo 100-0004, Japan

## Abstract

Despite the discovery of heterotrimeric αβγ G proteins ∼25 years ago, their selective perturbation by cell-permeable inhibitors remains a fundamental challenge. Here we report that the plant-derived depsipeptide FR900359 (FR) is ideally suited to this task. Using a multifaceted approach we systematically characterize FR as a selective inhibitor of Gq/11/14 over all other mammalian Gα isoforms and elaborate its molecular mechanism of action. We also use FR to investigate whether inhibition of Gq proteins is an effective post-receptor strategy to target oncogenic signalling, using melanoma as a model system. FR suppresses many of the hallmark features that are central to the malignancy of melanoma cells, thereby providing new opportunities for therapeutic intervention. Just as pertussis toxin is used extensively to probe and inhibit the signalling of Gi/o proteins, we anticipate that FR will at least be its equivalent for investigating the biological relevance of Gq.

## Abstract (web summary)

Pertussis toxin is used extensively for perturbing Gαi/o pathways in the study of physiology and disease, but an equivalent inhibitor of Gαq signalling is not currently available to the research community. Here the authors characterize FR900359 as a specific Gq inhibitor and demonstrate its utility to dissect GPCR signalling and its potential to inhibit melanoma cells.

## Graphical abstract

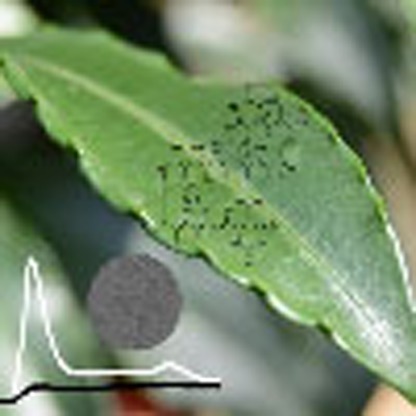

Many extracellular stimuli propagate cellular activity via G protein-coupled receptors (GPCRs), the largest family of cell surface signalling molecules comprising ∼800 members in humans^1^,^2^. Four families of heterotrimeric αβγ guanine nucleotide-binding proteins (G proteins) located at the cytoplasmic face of the plasma membrane suffice to receive, interpret and route these signals to diverse sets of downstream target proteins^3^,^4^,^5^,^6^,^7^,^8^. Thus, the mammalian GPCR-G protein signalling axis evolved to converge at the interface of receptor and G protein to then diverge at the interface of G proteins and effectors.

The mainstays of current pharmacotherapies are receptor agonists or antagonists, but conditions with complex pathologies such as cancer or pain, that involve multiple receptors and their associated signalling pathways, may be treated by manipulation of signalling at the post-receptor level^9^,^10^. Thus, pharmacological efficacy may be gained by targeting convergence points in signalling cascades downstream of activated receptors. Heterotrimeric G proteins are the first step in the GPCR signalling axis immediately downstream of activated receptors and are precisely the type of convergence points that would enable bypassing receptor diversity for the sake of increased pharmacological efficacy.

Although G proteins are of prime importance for maintaining homoeostasis in response to extracellular cues, no pharmacological agent that would enable a therapeutic grip on this protein family has become available since their discovery. Thus, heterotrimeric G proteins of all four subclasses (Gs, Gi/o, Gq/11 and G12/13) may be perceived as undruggable despite numerous cavities evident from X-ray crystallography that could be targets for pharmacological intervention^8^,^11^. YM254890 (YM), a cyclic depsipeptide of bacterial origin, co-crystallized together with its target protein Gq, provided the first high-resolution structure of a G protein-inhibitor complex^12^. Unfortunately, YM has been withdrawn by Astellas Pharma Inc. and is no longer available to researchers. Also, inaccessible is the bacterial strain *Chromobacterium* sp. QS3666 because it has not been deposited in a public culture collection.

An alternative to YM, readily accessible to the scientific community, is therefore needed urgently and would be of great value to understand the contribution of Gq signalling in physiology and disease, but also as a potential therapeutic target. Here we propose that FR900359 (FR, previous commercial name UBO-QIC, Fig. 1a) is such an alternative. Although first isolated in 1988 from the leaves of the ornamental plant *Ardisia crenata*^13^, its specificity for individual Gα isoforms is unknown and has never been studied systematically. Consequently, it is difficult to interpret the biological effects obtained with this reagent. We characterize FR and its G protein selectivity profile with the entire set of mammalian Gα isoforms, and identify its molecular mechanism of action using a panel of cell-based assays, purified proteins and molecular dynamics simulations. We demonstrate the analytical value of FR for the study of Gq activity in complex signalling networks, both in living cells and in an *ex vivo* model of Gq-mediated vasoconstriction. Importantly, we also demonstrate that FR does not affect signalling and basic cell functions when Gα_q_ and Gα_11_ have been deleted by CRISPR-Cas9 genome editing. Finally, we use FR to investigate the role of Gq proteins in cancer cells using melanoma as a model system. Our results reveal that silencing of Gq proteins rather than their linked receptors may be an innovative yet underappreciated molecular intervention to target oncogenic signalling at the post-receptor level.

## Results

### FR is Gq selective in second messenger assays

**Figure 1:**
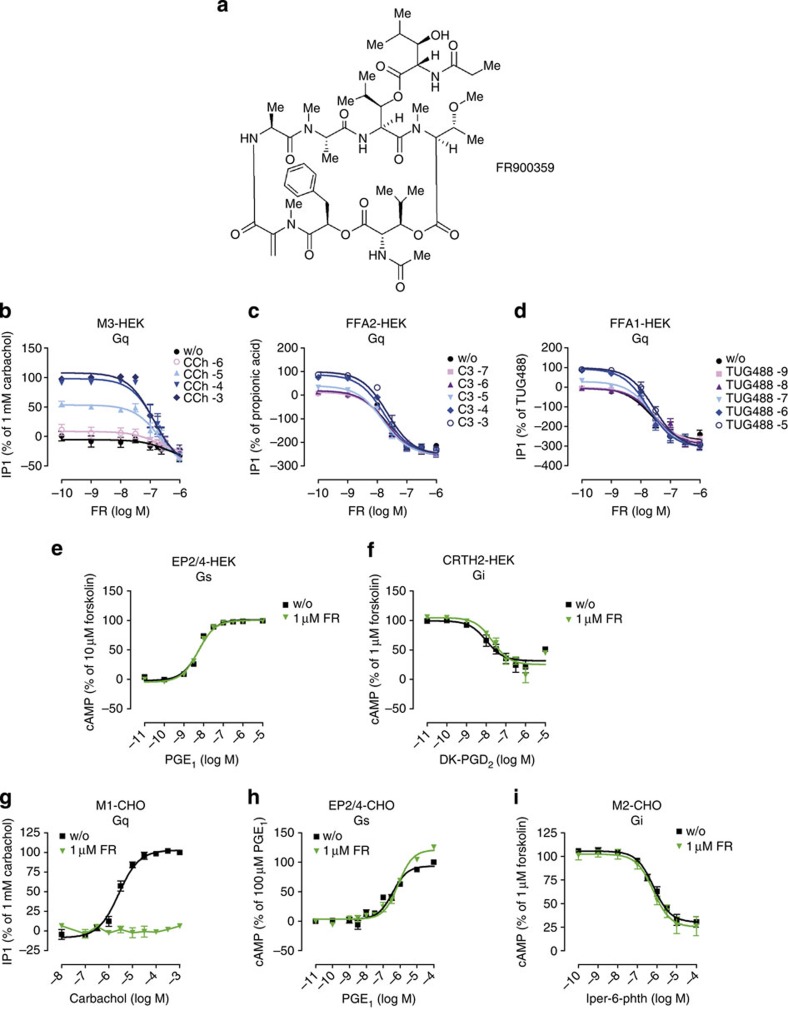
FR interdicts Gα_q_-dependent second messenger production in mammalian cell lines. (**a**) Chemical structure of the cyclic depsipeptide FR isolated from the leaves of *A. crenata*. (**b**–**d**) Agonist-induced IP1 production in HEK293 cells subsequent to stimulation of (**b**) endogenous muscarinic M3 receptors with CCh, (**c**,**d**) recombinant free fatty acid FFA2 and FFA1 receptors with propionic acid (C3) or small molecule FFA1 agonist TUG488, respectively, in absence and presence of various concentrations of FR. (**e**) Prostaglandin E_1_ (PGE_1_)-induced increase in intracellular cAMP in HEK293 cells with and without 1 μM FR. (**f**) 13,14-dihydro-15-keto-prostaglandin D_2_ (DK-PGD_2_)-induced decrease in intracellular cAMP in CRTH2-HEK293 cells in presence or absence of 1 μM FR. (**g**,**h**) Agonist-induced second messenger production in CHO cells with or without FR (1 μM). (**g**) Elevation of intracellular IP1 mediated by Gq-linked M1 receptors stimulated in response to CCh. (**h**) PGE_1_-induced increase of intracellular cAMP via stimulation of endogenous, Gs-sensitive prostaglandin EP2/4 receptors. (**i**) Iper-6-phth-mediated decrease in intracellular cAMP via Gi-linked muscarinic M2 receptors in M2-CHO cells. (**b**–**i**) Data are means±s.e.m. of *n*=2–8 experiments conducted at least in triplicate.

We purified FR (Fig. 1a) by activity-guided fractionation of *A. crenata* leaf extracts. Although FR is structurally closely related to YM (Supplementary Fig. 1), we cannot rule out that subtle structural differences may result in divergent functional activities. Accumulation of inositol monophosphate (IP1) is an established measure of Gq-coupled signalling to phospholipase Cβ (PLCβ) isoforms^14^. Therefore, FR was initially assessed for its capacity to blunt IP1 production in HEK293 cells on stimulation of three distinct Gq-linked receptors (muscarinic M3 endogenously expressed and free fatty acid receptors FFA1 and FFA2, forcibly expressed in this cell system). Consistent with Gq inhibition, ligand-mediated IP1 accumulation was completely suppressed by FR in a concentration-dependent manner (Fig. 1b–d). Inhibition profiles were non-competitive, independent of the chosen Gq-sensitive receptor and the extent of basal receptor activity that was low in native HEK293 cells but highly apparent when constitutively active FFA1 and FFA2 were overexpressed (Fig. 1b–d and Supplementary Fig. 2). FR concentrations sufficient to fully block Gq-mediated IP1 accumulation, did not perturb the cAMP-raising by Gs-sensitive prostanoid EP2/EP4 receptors (Fig. 1e) or cAMP lowering by Gi-sensitive prostaglandin D2 receptor DP2/CRTH2 (hereafter CRTH2; Fig. 1f). An essentially identical selectivity profile was obtained when Gq-, Gs- and Gi-linked receptors were examined in Chinese hamster ovary (CHO) cells as recombinant host (Fig. 1g–i).

Because FR is of plant origin, we wondered whether plants use this molecule to regulate their own G protein signalling. Plant G proteins are self-activating^15^, therefore we took advantage of this property and monitored spontaneous GDP release of a plant Gpa1 in the absence and presence of FR. We found that concentrations of FR tenfold in excess of those sufficient to fully blunt mammalian Gq signalling hardly impacted the kinetics of Gpa1 nucleotide exchange (Supplementary Fig. 3). Consistent with this observation, FR did not alter Gpa1 thermal stability as assessed by fast quantitative cysteine reactivity (Supplementary Fig. 4). Together, our results suggest that FR is highly selective for inhibition of mammalian Gq signalling and that this effect is cell-type independent.

### Selectivity of FR across all mammalian Gα isoforms

**Figure 2:**
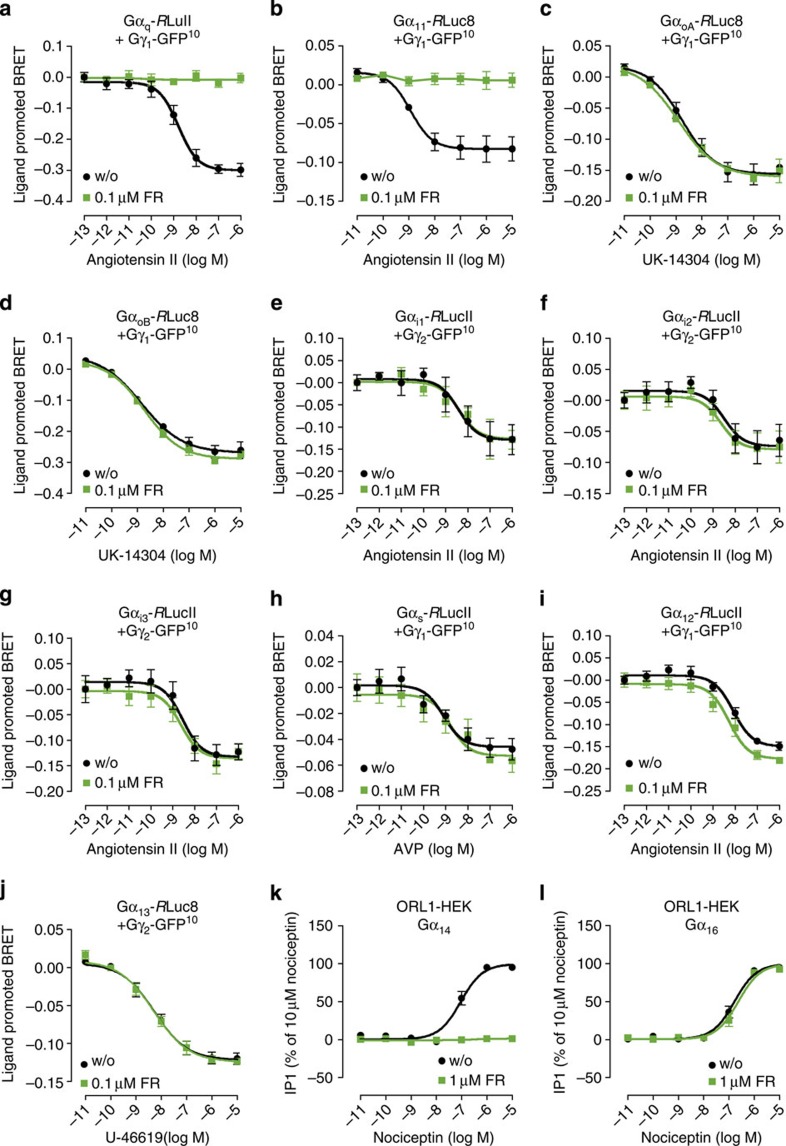
FR inhibits Gα_q_, Gα_11_ and Gα_14_, but not Gα_16_ or any other mammalian Gα isoform. (**a**–**j**) BRET experiments conceived with Gα subunits fused to energy donor *R*LucII or *R*Luc8, respectively, and Gγ subunits fused to energy acceptor GFP^10^ in HEK293 cells transiently transfected to coexpress the indicated BRET probes. FR completely inhibits the activation of (**a**) Gα_q_-, and (**b**) Gα_11_-containing heterotrimers induced upon stimulation with angiotensin II via coexpressed AT1 receptors. In (**c**–**j**) BRET recordings were obtained by coexpressing adrenergic α2_C_-AR (**c**,**d**), angiotensin II AT1 receptor (**e**–**g**,**i**), arginine vasopressin V2 receptor (**h**), and thromboxane TPα receptor (**j**). Cells were initially pretreated with FR (0.1 μM) or solvent and then stimulated with the indicated agonists. (**k**,**l**) IP1 accumulation in HEK293 cells reconstituted by transient coexpression of the nociceptin ORL1 receptor along with Gα_14_ or Gα_16_, respectively, in absence and presence of 1 μM FR. Data are means±s.e.m. of *n*=3–6 independent experiments conducted at least in triplicate.

While second messenger assays are well-suited to determine selectivity of G protein inhibitors for the major Gα subfamilies, they cannot discriminate between individual Gα isoforms. To provide an accurate and comprehensive characterization of FR selectivity we chose an experimental approach based on bioluminescence resonance energy transfer (BRET) that relies on co-transfection of GPCRs with an extended panel of Gα subunit sensors. In particular, we applied Gα_s_, Gα_i1_, Gα_i2_, Gα_i3_, Gα_oA_, Gα_oB_, Gα_q_, Gα_11_, Gα_12_, Gα_13_ fused to either *R*LucII or *R*Luc8, a more sensitive BRET^2^ energy donor^16^, along with Gγ_2_-GFP^10^ as energy acceptor and unlabelled Gβ_1_. This sensor combination has proven valuable to monitor activation-associated conformational changes within G proteins that occur upon receptor stimulation^17^,^18^. HEK293 cells transiently transfected to express individual BRET probes along with GPCRs from different coupling classes responded with agonist-dependent BRET decreases that were concentration dependent and well-suited to quantify activation of all Gα isoforms (Fig. 2a–j). When cells were pretreated with FR, agonist BRET responses of Gα_q_- and Gα_11_-containing heterotrimers were completely prevented (Fig. 2a,b) but those of all other Gα isoforms were not (Fig. 2c–j). As Gα-*R*Luc probes were not available for Gq family members Gα_14_ and Gα_16_, we took advantage of their ‘promiscuity' to link non-Gq-GPCRs to stimulation of the Gq-effector PLCβ (refs 19, 20) and thereby production of IP1. HEK293 cells are an ideal host to reconstitute such functional interaction as they express Gα_q_ and Gα_11_ but not Gα_14_ or Gα_16_ (ref. 21). Indeed, both Gα_14_ and Gα_16_ translated activation of the Gi-sensitive ORL1 receptor into robust accumulation of IP1 on treatment with the cognate agonist nociceptin (Fig. 2k,l). Yet, only ORL1-G14 but not -G16 interaction was fully prevented by FR (Fig. 2k,l). Nociceptin was ineffective in cells transfected with empty vector, ORL1 or the respective Gα proteins alone (Supplementary Fig. 5) confirming the specificity of the ‘reconstitution' experiment. We further interrogated whether inhibition by FR may be more specific to any of the sensitive isoforms and quantified its capacity to blunt signalling of individual Gq family members (Supplementary Fig. 6). We found that inhibition of Gα_q_, Gα_11_ and Gα_14_ signalling by FR similarly required low micromolar concentrations. In contrast, even in the presence of excess FR signalling via Gα_16_ was completely unaffected. These results illustrate that FR is highly selective for inhibition of Gα_q_, Gα_11_ and Gα_14_, and interdicts signal transduction by acting directly on the G protein but not the GPCR.

### FR does not impair G protein-independent β-arrestin recruitment

**Figure 3:**
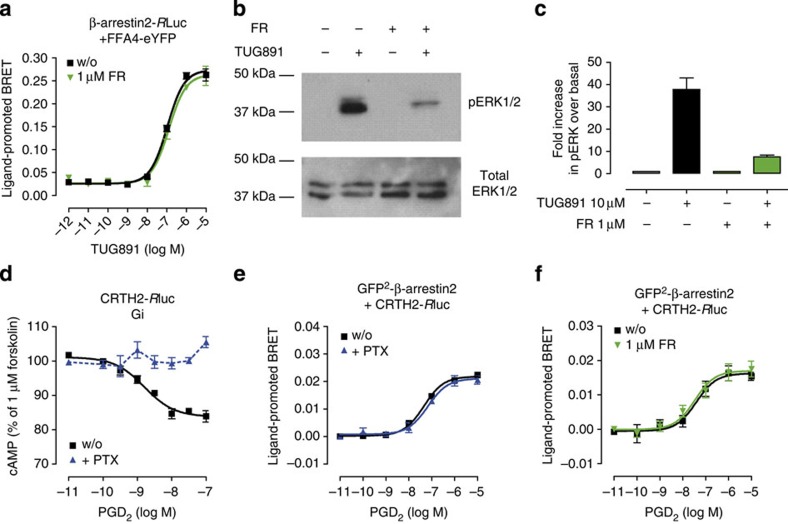
FR interdicts Gq-dependent ERK1/2 activation but not β-arrestin recruitment. (**a**) BRET-based β-arrestin recruitment assay between energy donor β-arrestin2-*R*Luc and energy acceptor FFA4-eYFP transiently coexpressed in HEK293 cells upon treatment with FFA4 agonist TUG891 in presence and absence of 1 μM FR. (**b**) pERK1/2 response induced by stimulation with TUG891 (10 μM) for 5 min is largely diminished but not eliminated by FR (1 μM). (**c**) Quantification (bar graphs) of pERK1/2 after stimulation with TUG891 (10 μM) for 5 min using the Alphaimager software. (**d**) Gi inhibitor PTX fully prevented receptor-mediated inhibition of cAMP by Gi-sensitive CRTH2 in CRTH2-*R*Luc-GFP^2^-β-arrestin2-HEK293 cells. (**e**,**f**) prostaglandin D_2_ (PGD_2_)-induced β-arrestin recruitment conceived with CRTH2-*R*Luc and β-arrestin2-GFP^2^ is insensitive to pretreatment with (**e**) PTX and (**f**) FR. (**a**,**c**–**f**) Means +/± s.e.m. of *n*=2–12 experiments performed at least in triplicate, (**b**) representative western blot that was repeated three times.

β-arrestin proteins, known for their capacity to desensitize and downregulate GPCR signalling^22^ also act as multiprotein scaffolds that orchestrate proximity and function of signalling molecules downstream of activated GPCRs^23^,^24^. β-arrestin recruitment to activated receptors may occur both subsequent to or independent of heterotrimeric G protein engagement^24^,^25^. Free fatty acid receptor FFA4 and prostaglandin D_2_ receptor CRTH2 are two examples for Gq- and Gi-linked receptors, respectively, where G protein activation and β-arrestin recruitment operate independent from each other^26^,^27^. Consistent with this notion, Gq-independent β-arrestin recruitment by FFA4 was entirely insensitive to FR pretreatment (Fig. 3a), but activation of extracellular signal-regulated kinases 1 and 2 (ERK1/2), a cellular event known to be largely Gq-mediated^28^, was not (Fig. 3b,c). In fact, FFA4-CHO cells pretreated with FR displayed diminished ERK activation at all doses of FFA4 agonist TUG891 (Supplementary Fig. 7). Consistent with these findings, Gq-dependent IP1 accumulation was also entirely blunted by FR (Supplementary Fig. 8). Similarly, pertussis toxin (PTX) fully prevented CRTH2-dependent lowering of cAMP (Fig. 3d), but was without effect on Gi-independent β-arrestin recruitment triggered with the cognate agonist for this receptor (Fig. 3e). Again, FR did not interfere with this G protein-independent cellular event (Fig. 3f), highlighting its capacity to discriminate between Gq-dependent and β-arrestin-promoted cell behaviours.

### FR unmasks Gq signalling in whole-cell activation profiles

**Figure 4:**
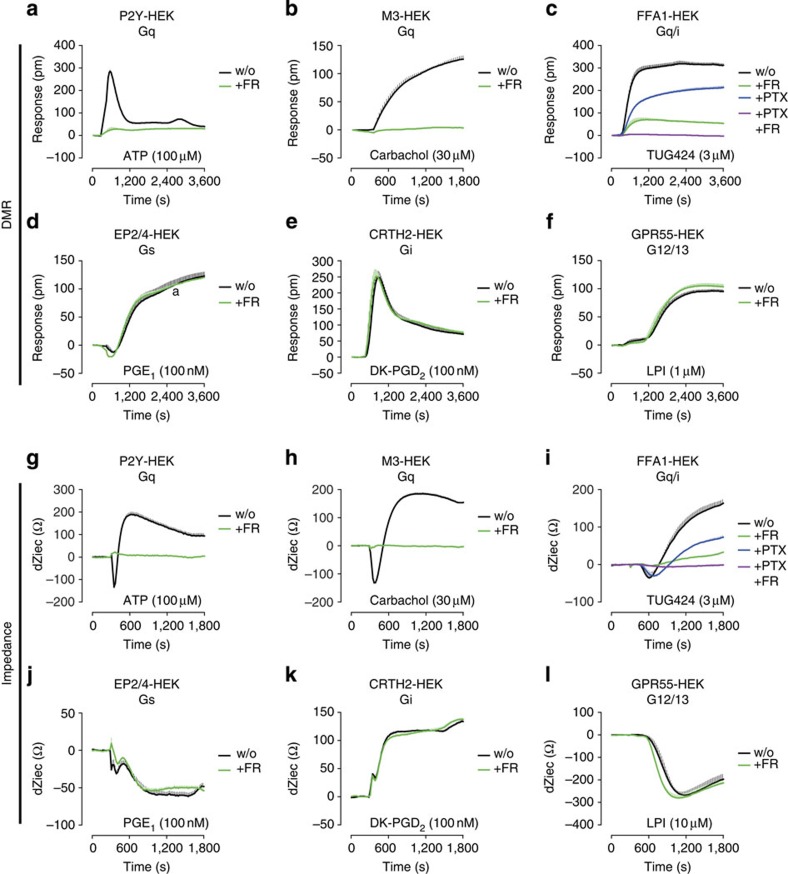
Label-free DMR establishes specificity of FR for inhibition of Gq over Gs, Gi, and G12/13 proteins. (**a**–**f**) DMR, quantified as changes in reflected wavelength (Δpm) or (**g**–**l**) bioimpedance, quantified as chances in the extracellular current (dZiec), induced by the indicated agonists in native or recombinant HEK293 cell lines, pretreated or not with Gq inhibitor FR (1 μM), Gi inhibitor PTX (50 ng ml^−1^), or both. (**a**,**b**) DMR recordings of endogenous Gq-selective (**a**) P2Y or (**b**) muscarinic M3 receptors obtained upon stimulation with ATP or CCh, respectively. (**c**) DMR profile of the promiscuous Gq/i-linked FFA1 receptor in FFA1-HEK293 cells by small molecule agonist TUG424. (**d**–**f**) DMR profiles reflecting (**d**) Gs, (**e**) Gi and (**f**) G12/13 pathway engagement by the stimuli PGE_1_ for endogenous EP2/4, DK-PGD_2_ for recombinant CRTH2, and lysophosphatidylinositol for recombinant GPR55, respectively. (**g**–**l**) Experiments equivalent to those depicted in **a**–**f** but obtained with bioimpedance whole-cell sensing (Cellkey). Data shown are representative traces (means+s.e.m.) from *n*=4 experiments conducted in triplicate. w/o, without.

An intrinsic property of many activated GPCRs is their initiation of multiple signalling waves via engagement of heterotrimeric G proteins and/or β-arrestins, respectively, a phenomenon referred to as coupling multiplicity or coupling pleiotropy^24^,^29^. In this regard holistic label-free detection platforms based on dynamic mass redistribution (DMR) or bioimpedance may be advantageous, because they provide unbiased, real-time kinetic resolution of complex GPCR signalling in living cells^30^,^31^. We initially investigated the consequences of pharmacological Gq inactivation on integrated cell responses of GPCRs from all four coupling classes including one with dual pathway coverage. DMR response profiles elicited by ATP and carbachol (CCh), which stimulate endogenous Gq-linked P2Y and muscarinic M3 receptors, respectively, were virtually abolished by FR (Fig. 4a,b). In contrast, activity traces triggered by TUG424 via the Gq/Gi-linked FFA1 receptor were only abrogated when FR was combined with the Gi inhibitor PTX (Fig. 4c). Stimuli acting via *bona fide* receptors for Gs-, Gi- and G12/13-mediated signalling were unaffected by pretreatment with FR (Fig. 4d–f). Essentially similar findings were obtained when these experimental settings were recapitulated using bioimpedance-based cell sensing (Fig. 4g–l). FR, on its own, did not elicit detectable cell activation (Supplementary Fig. 9) and stimuli chosen for Gs, Gi and G12/13 engagement were validated to reflect the signalling pathways previously assigned to them (Supplementary Fig. 10, also see ref. 30 for comprehensive validation of receptor signatures with antagonists, pathway modulators and second messenger assays). From these results we infer that FR is highly selective for inhibition of Gq-dependent signalling outcomes and well-suited to investigate contribution of Gq input to complex cell-activation profiles.

### FR is inactive in cells lacking Gα_q/11_

**Figure 5:**
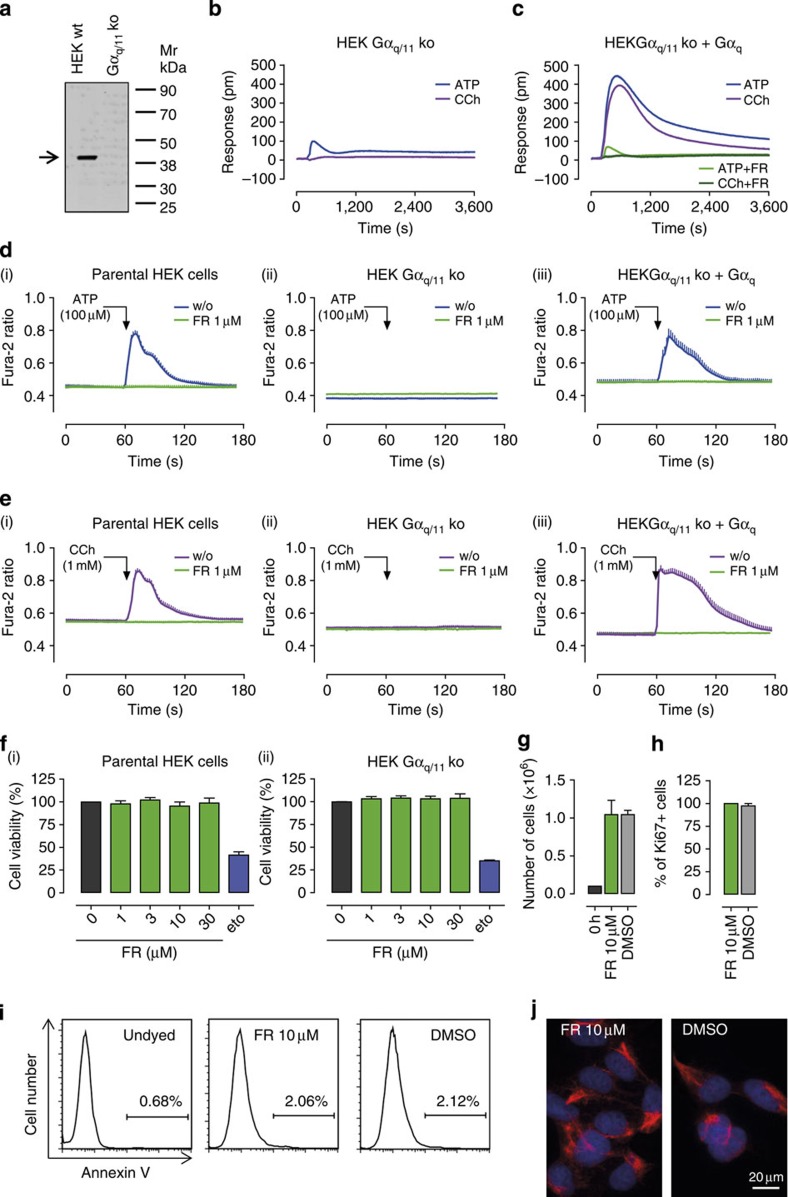
FR does not exert cellular effects that are independent of Gα_q_ and Gα_11_. (**a**) Western blot analysis of Gα_q/11_ expression in HEK293 wild-type cells (left) and HEK293 cells in which genes for Gα_q/11_ were inactivated by CRISPR-Cas9-mediated genome editing (HEK293 Gα_q/11_ ko cells, right). Black arrow: size of Gα_q/11_ (40 kDa). (**b**,**c**) DMR induced by ATP (100 μM) or CCh (30 μM) in HEK293 Gα_q/11_ knockout cells transiently transfected with either (**b**) pcDNA3.1(+) or (**c**) Gα_q_ with and without pretreatment with 1 μM FR (green traces in **c**). (**d**) ATP- or (**e**) CCh-mediated release of intracellular calcium in single cells without (blue traces) or after pretreatment with FR 1 μM for 30 min (green traces) in (i) parental HEK293 wild-type cells, (ii) HEK293 Gα_q/11_ knockout cells and (iii) HEK293 Gα_q/11_ knockout cells transfected to express Gα_q_.(**f**) Cell viability of (i) HEK293 wild type and (ii) HEK293 Gα_q/11_ knockout cells assessed in a cytotoxicity assay applying resazurin in presence of various supramaximal concentrations of FR. The cytotoxic anticancer drug etoposide (eto) 50 μM was used as a positive control. (**g**) Cell growth and (**h**) expression of the cellular proliferation marker Ki67 in HEK293 Gα_q/11_ knockout cells (**g**) 72 h or (**h**) 24 h after cells were treated with FR 10 μM or 0.1% DMSO. (**i**) Flow cytometry analysis of HEK293 Gα_q/11_ knockout cells using the apoptosis marker annexin V after pretreatment with FR 10 μM or DMSO 0.1% for 72 h. (**j**) Cellular distribution of intracellular vimentin filaments in HEK293 Gα_q/11_ knockout cells 24 h after pretreatment with FR 10 μM or DMSO 0.1%. red, vimentin; blue, Hoechst; bar, 20 μm. (**a**–**e**,**i**,**j**) One representative experiment that was repeated at least twice. (**f**–**h**) Mean values+s.e.m. of at least two independent experiments.

To further corroborate specificity of FR action in phenotypic assays we examined its capacity to affect signalling and function in HEK293 cells lacking functional Gq proteins by CRISPR-Cas9-mediated genome editing. We targeted the genes coding for Gα_q_ and Gα_11_ because, among the four members of the Gα_q_ family, Gα_q_ and Gα_11_, but not Gα_14_ and Gα_16_, are expressed in HEK293 cells^21^. Consistent with the absence of Gα_q/11_ as determined by western blot analysis (Fig. 5a), *bona fide* Gq-stimuli ATP and CCh were markedly impaired (ATP) or completely inactive (CCh) in holistic DMR recordings on Gα_q/11_ knockout cells (Fig. 5b). However, reintroduction of Gα_q_ into the knockout background was required and sufficient to fully restore both, agonist responses and their inhibition by FR (Fig. 5c). Essentially equivalent results were obtained when the above stimuli were applied in single-cell Ca^2+^ recordings (Fig. 5d,e). On its own, FR did not alter global cell function irrespective of the presence or absence of Gα_q/11_ (Supplementary Fig. 11). To further assess potential off-target activity of FR we analysed cell viability in parental and Gα_q/11_ knockout cells exposed to FR. We find that FR does not disturb cellular metabolic homoeostasis even at concentrations by far exceeding those to silence Gα_q/11_ signalling (Fig. 5f). Absence of Gq-independent effects were also evident from phenotypic assays examining alterations of basic cellular functions in cells lacking Gα_q/11_. FR did not alter cell growth (Fig. 5g), the percentage of proliferating cells as determined by staining for the nuclear cell cycle marker Ki67 (Fig. 5h), apoptosis rate as quantified by life cell Annexin V staining (Fig. 5i) or distribution of vimentin filaments in the cytoplasm as potential indicator of morphological abnormalities (Fig. 5j). Together, knockout and rescue experiments along with the lack of measurable phenotypes in response to FR using cells with Gα_q/11_ deletion, unambiguously prove that inhibition of cellular functions by FR is mediated via direct targeting of Gq.

### Action of FR in a model of Gq-mediated vasoconstriction

**Figure 6:**
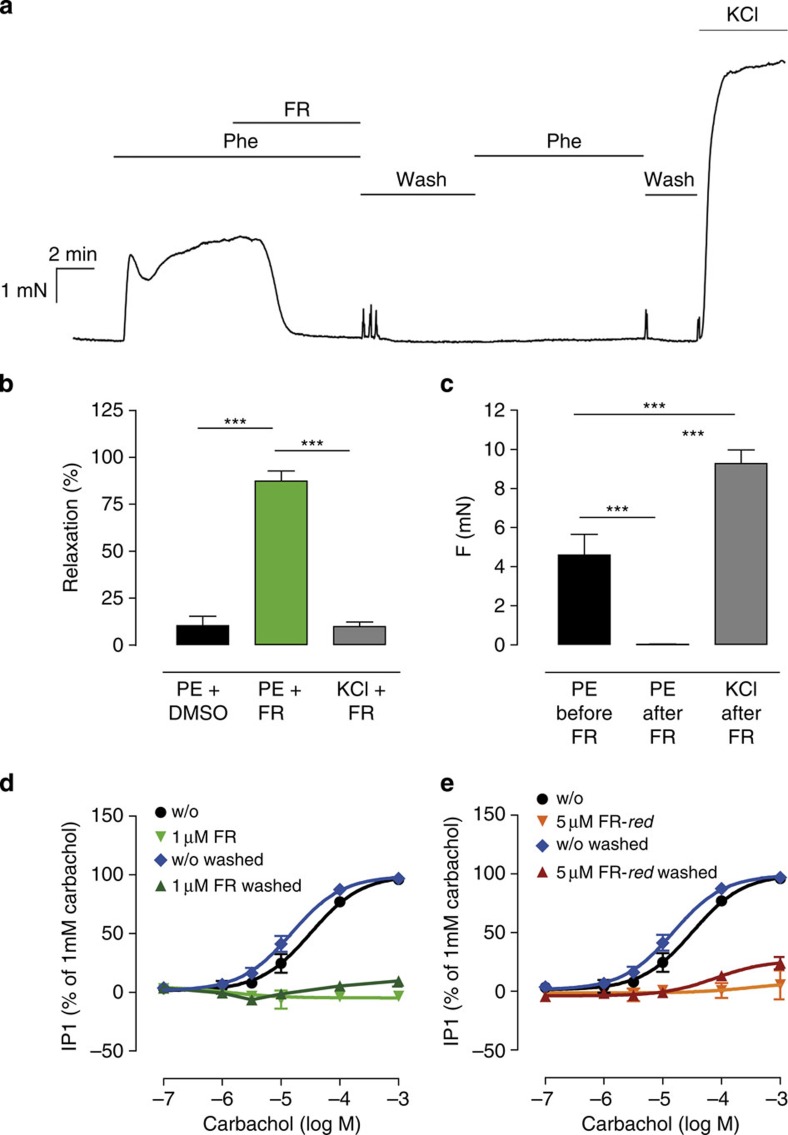
FR is a powerful, yet pseudo-irreversible relaxant of mouse tail arteries after Gq-dependent vasoconstriction. (**a**) Original trace of isometric force measurement in mouse tail arteries using α_1_AR agonist PE (0.5 μM) for submaximal preconstriction and FR (1 μM) for relaxation. (**b**) Quantification and statistical analysis of vasorelaxation after PE-mediated preconstriction by DMSO (mean±s.e.m.=10.3±5.0%, *n*=12) and FR (mean±s.e.m.=87.4±5.3%, *n*=12), or after KCl (30 mM) constriction by FR (mean±s.e.m.=9.8±2.4%, *n*=6). (**c**) Statistical analysis revealing irreversibility of FR response: PE-induced vasoconstriction before (mean±s.e.m.=4.6±1.0 mN, *n*=7) and after FR pretreatment (mean±s.e.m.=0.01±0.04 mN, *n*=7) including repeated washing steps; KCl-induced constriction after FR pretreatment (mean±s.e.m.=9.3±3.5, *n*=7). (**d**,**e**) muscarinic M3 receptor-mediated production of IP1 induced by CCh in HEK293 cells in presence and absence of (**d**) FR and (**e**) its reduced form ‘FR-*red*' prepared by catalytic hydrogenation, with or without washout. (**a**) A representative isometric force measurement that was repeated 6–12 times. (**b**,**c**) Means+s.e.m. of *n*=6–12 measurements. ***P<0.001 according to a one-way analysis of variance and subsequent Tukey's *post hoc* test. (**d**,**e**) Means±s.e.m. of *n*=3–6 experiments performed at least in duplicate.

We next sought to expand our studies on FR action from cells to more complex and physiologically relevant organ systems. Many potent vasoconstrictors such as norepinephrine, endothelin or angiotensin elicit their effects via Gq-GPCRs, thereby contributing to the regulation of vascular tone and blood pressure. We focused on phenylephrine (PE), an α_1_ adrenergic receptor (α_1_AR) agonist, previously thought to modulate vascular tone via interaction with Gq/11 proteins and their downstream signalling components^32^. Specifically, we investigated whether pharmacological Gq inactivation is required and sufficient to counteract PE-dependent increase of vascular tone using *ex vivo* preparations of mouse tail arteries, an established model for physiologically relevant resistance arteries. When arteries were submaximally pre-constricted with PE, FR but not its solvent strongly reduced vascular tone (Fig. 6a,b). On the contrary, FR was essentially inactive when tail arteries were submaximally pre-constricted with the receptor- and Gq-independent vasoconstrictor KCl (Fig. 6b,c), confirming the Gq-specific nature of the observed relaxation. Interestingly, vascular relaxation by FR was apparently irreversible because PE-mediated constriction did not re-emerge even after extensive washout of FR (Fig. 6a,c). This lack of PE-effect is not due to deterioration of the preparations because vessels remained responsive to the Gq-independent vasoconstrictor KCl (Fig. 6a,c). Our findings validate PE-dependent, α_1_AR-mediated vasoconstriction as an *ex vivo* Gq paradigm and nicely illustrate the value of FR as a powerful pharmacological agent to unmask Gq contribution even in more complex *ex vivo* settings.

### FR inhibits activation of Gq in a pseudo-irreversible manner

Resistance to washout of FR-dependent vasorelaxation may be indicative of covalent or at least very tight interaction between FR and its target Gq. Alternatively, FR may accumulate near its target or be hindered in free diffusion away from its target in the complex *ex vivo* setting. Indeed, FR carries an α,β-unsaturated carbonyl group of an *N*-methyldehydroalanine (*N*-MeDha) residue, which may be susceptible to nucleophilic attack, for instance via Michael addition (Supplementary Fig. 12a). Thus, it is conceivable that FR inhibition of Gq signalling could occur via covalent interaction. We tested this hypothesis in intact cells that lack the complexity of intact vessels, where microanatomic properties of effect compartments may confound mechanistic analysis. We incubated HEK293 cells with FR for 1 h, followed by extensive washing before activation of endogenous Gq-sensitive muscarinic M3 receptors with CCh. Interestingly, Gq inhibition was also retained after washout even in this less complex cell-based experiment (Fig. 6d) indicating formation of a covalent complex or, alternatively, very tight association of FR with Gq. To discriminate between these possibilities, we abolished the reactive moiety by catalytic hydrogenation to afford a reduced form of FR (Supplementary Fig. 12a, hereafter ‘FR-*red*'). FR-*red* still displayed potent Gq inhibition (Supplementary Fig. 12b), and this effect remained after washout (Fig. 6e). These results suggest that the α,β-unsaturated carbonyl group within FR is not crucial for Gq/11 inhibition and that FR and Gq do not interact in a covalent way. We therefore posit that a slow off-rate may contribute significantly to the high ‘affinity' of FR to Gq, accounting for both pseudo-irreversible inhibition and resistance to washout in *ex vivo* vessel preparations as well as in intact cells in culture.

### Docking and molecular dynamics (MD) simulations of FR

**Figure 7:**
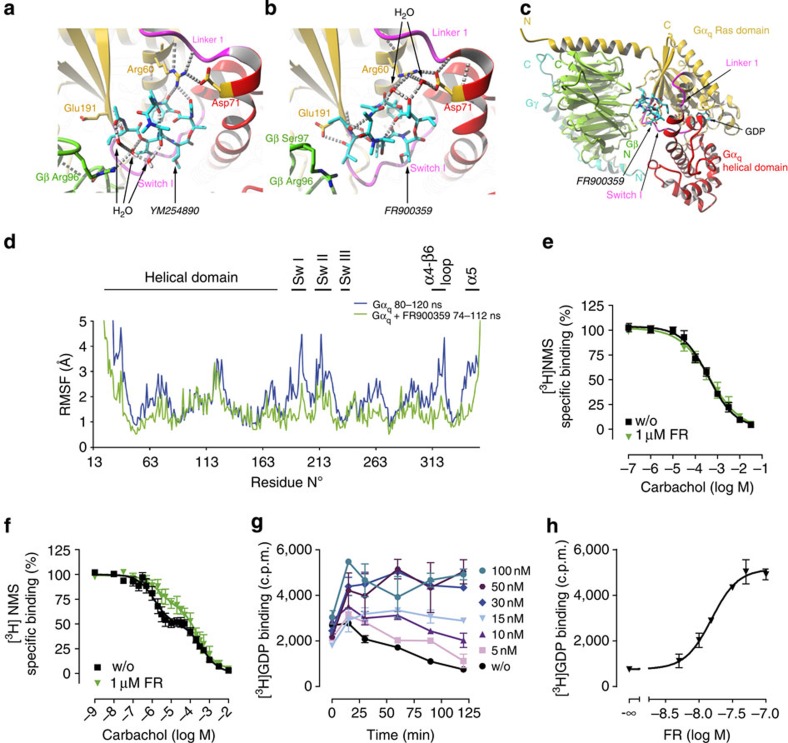
FR inhibits Gq by acting as GDI. (**a**,**b**) Interaction of YM ((**a**), pdb 3ah8 (ref. 22)) and FR ((**b**), derived from docking) with the switch I and linker 1 region of Gα_q_ (both coloured magenta). Hydrogen bonds are presented as grey dashed lines. Interacting key residues are shown as stick model. Gα_q_ subunits and domains are colour coded: Gα_q_ Ras domain: yellow, helical domain: red, Gβ: green. (**c**) Zoomed-out view of the heterotrimeric Gq protein bound to FR. (**d**) All-atom molecular dynamics simulations to estimate average r.m.s. fluctuations of Gα_q_ and Gα_q_+FR. (**e**,**f**) [^3^H]NMS displacement by CCh in **e** whole-cell binding assays and (**f**) M1-CHO membranes in presence or absence of FR (1 μM). (**g**) [^3^H]GDP dissociation from purified Gα_q_ proteins in presence of different concentrations of FR. (**h**) Quantification of [^3^H]GDP dissociation in presence of FR estimated as [^3^H]GDP binding to Gα_q_ after 120 min dissociation. (**e**–**h**) Means±s.e.m. of *n*=3–6 experiments performed at least in duplicate.

Comprehensive *in vitro* characterization using pathway-unbiased and signal-specific endpoint assays suggest that FR shares several hallmark features with YM, such as selectivity for inhibition of Gα_q/11/14_ isoforms^12^ or non-covalent interaction with Gq^33^, yet information on its molecular mechanism of action is lacking. YM has recently been co-crystallized in complex with Gα_q_-βγ thereby providing atomic level insight into its mode of action at a molecular level^12^. We took advantage of this information in combination with molecular docking studies to investigate whether structural differences between FR and YM might impact their binding poses within the Gq protein. Binding energies for both inhibitors determined through independent docking runs were very similar (9.76 kcal mol^−1^ versus 9.85 kcal mol^−1^ for FR and YM, respectively) and the best scoring docking result of Gq-bound FR aligned well with that previously determined for YM in complex with Gq (Fig. 7a,b, for zoomed-out versions of G protein-inhibitor complexes see Fig. 7c and Supplementary Fig. 13). Consistent with these findings, all-atom MD simulations, which build on information from crystal structures to model protein movements in solution^15^,^34^, revealed that FR occupancy of Gα_q_ reduced local flexibility in switch region I and II and also did so very efficiently in the α4-β6 loop and C-terminal α5 helix, regions known to be crucial for receptor-mediated G protein activation^8^,^11^,^35^ (Fig. 7d and Supplementary Fig. 14 for colour coding of all Gα_q_ regions with impaired domain motions). Movement of Gα switch regions is critical to commencement of G protein signalling^8^,^11^,^36^ and impaired ‘switch movement' would also be entirely consistent with inhibition of GDP release. We therefore infer that FR likely functions as guanine nucleotide dissociation inhibitor (GDI), that is, shares its principal mode of action with YM.

### The molecular mechanism of FR action

To test the predictions from our computational studies we performed binding assays on M1-CHO cells or membranes isolated from these cells that express the Gq-activating muscarinic M1 receptor. Whole-cell binding assays can tell whether or not inhibitors of receptor function act via perturbation of agonist binding. Membrane-binding assays further advance mechanistic insight because they allow distinction between GDIs and GTP entry inhibitors^37^. In whole cells, FR did not affect CCh displacement of the radioantagonist [^3^H]*N*-methylscopolamine ([^3^H]NMS; Fig. 7e). These data illustrate that inhibition of Gq-GPCR function by FR is not due to perturbation of agonist binding. In M1-CHO membranes CCh displacement of [^3^H]NMS is biphasic because GPCR agonists bind with high affinity to a fraction of receptors that are also coupled to G proteins and with lower affinity to G protein-uncoupled receptors (Fig. 7f)^11^,^37^,^38^,^39^. These conditions can visualize GDI activity: GDIs specifically inhibit high-affinity agonist binding, because they preclude ternary complex formation, that is, short-lived ligand–receptor complexes that are stabilized by nucleotide-free G proteins. Indeed, while FR had no effect on the affinity of NMS to M1 receptors (Supplementary Fig. 15) the Gq inhibitor significantly diminished (by 40%) high-affinity CCh binding, indicating that it interferes with ternary complex formation of agonist, receptor and G protein (Fig. 7f). Unexpectedly, FR did not fully abolish high-affinity CCh binding that can be rationalized if (i) M1 is linked to G proteins from more than one family, (ii) intracellular adaptors other than G proteins promote high-affinity agonist binding, or (iii) FR does not act as GDI and therefore differs mechanistically from YM. To address this question, we quantified dissociation of tritiated GDP from purified recombinant Gα_q_ proteins that were isolated from Sf9 cells (Fig. 7g). High concentrations of FR completely prevented [^3^H]GDP dissociation with a half effective concentration of 15 nM (Fig. 7h and Supplementary Table 1 to demonstrate that [^3^H]GDP dissociation was independent of the time point chosen). This directly illustrates that FR acts as a potent GDI for Gα_q_.

### FR induces G1 cell cycle arrest in melanoma cells with Gq tone

**Figure 8:**
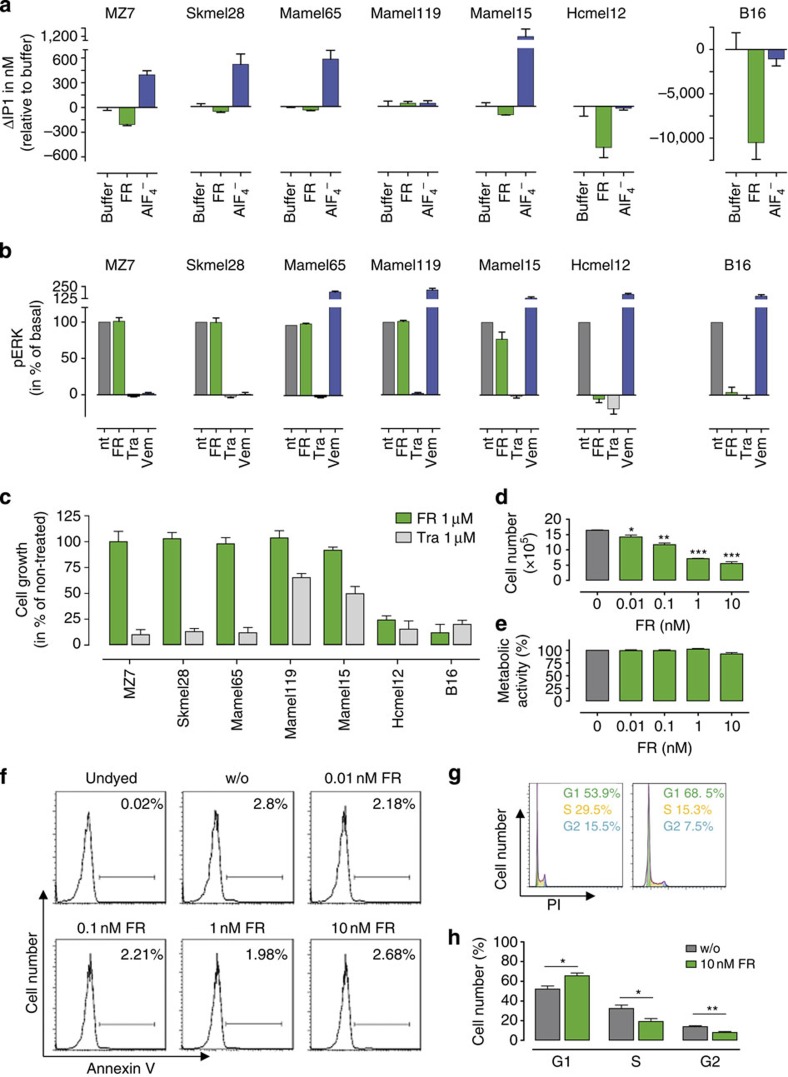
FR limits cell proliferation via G1 cell cycle arrest in melanoma cells with high Gq tone. (**a**) IP1 levels in the seven melanoma cell lines MZ7, Skmel28, Mamel65, Mamel119, Mamel15, Hcmel12 and B16 without or after pretreatment with 10 μM FR (Hcmel12) or 1 μM FR (all other melanoma cell lines) for 2 h. Blue bars show IP1 production stimulated by the pan-G protein activator AlF_4_^−^. Depicted are IP1 levels relative to buffer per 100,000 cells (MZ7, Skmel28, Mamel65, Mamel119, Mamel 15 and B16) or 10,000 cells (Hcmel12). (**b**) ERK phosphorylation in the indicated melanoma cells lines that were non-treated (nt), or pretreated with FR 1 μM, the MEK inhibitor Trametinib (Tra) 1 μM, or the specific B-Raf V600E inhibitor vemurafenib (Vem) 1 μM for 1 h, quantified in a HTRF-based ERK1/2 phosphorylation assay. Melanoma cells that do not harbour the mutated B-Raf V600E allele exhibit paradoxical ERK activation, consistent with published findings, for example, ref. 70. (**c**) Effect of FR (1 μM) and Tra (1 μM) on melanoma cell proliferation. (**d**) Effect of different concentrations of FR on the cell growth of B16 cells measured by trypan blue staining. (**e**) Metabolic activity of B16 cells using the XTT assay after 72 h of FR treatment. (**f**,**g**) Flow cytometry analysis of B16 cells using (**f**) the apoptosis marker annexin V and (**g**) propidium iodide (PI) as an indicator for intracellular DNA content in presence or absence of FR for 72 h. (**h**) Quantification of B16 cells in the G1, S and G2 phase of the cell cycle in presence or absence of FR (10 nM) for 72 h. (**d**) *P<0.05, **P<0.01, ***P<0.001 according to a two-tailed Student's *t*-tests. (**a**,**b**,**d**,**h**) Means+s.e.m. of *n*=2–5 experiments performed at least in duplicate. (**c**,**e**–**g**) One representative experiment that was at least repeated once.

Inhibition of Gq may represent an effective molecular intervention to target oncogenic signalling at a post-receptor convergence point, particularly for those tumour cells in which signs of elevated Gq activity are apparent. The latter may be induced by aberrant expression of wild-type and/or mutated Gq-GPCRs, as well as constitutively active Gα_q/11_ mutants such as Gα_q/11_R183C or Gα_q/11_Q209L, respectively, both considered as oncogenic drivers in melanomas of the eye and skin^40^,^41^. Forced enrichment of HEK293 cells with Gα_q_R183C and Gα_q_Q209L confers significant elevation of cell-intrinsic IP1 production and this effect was entirely blunted (Gα_q_R183C) or diminished (Gα_q_Q209L) by FR (Supplementary Fig. 16). We therefore applied FR as an indicator to identify melanoma cell lines with elevated Gq activity using both IP1 production and phosphorylation of extracellular signal-regulated kinases ERK1/2 (also named MAP kinases) as readouts. We found FR-sensitive reductions of basal signalling in B16, MZ7 and Hcmel12 but not in Mamel65, Mamel15, Mamel119 or Skmel28 cells (Fig. 8a,b; for mutational status of melanoma cells regarding mutant Gα_q/11_ alleles and key MAP kinase pathway drivers B-Raf and N-Ras, see Supplementary Table 2). MEK inhibitor, Trametinib (Tra) blunted ERK phosphorylation in all cell lines, and Vemurafenib (Vem) inhibited ERK activation only in cell lines harbouring the mutant B-RafV600E allele, entirely consistent with their associated modes of action (Fig. 8b). Interestingly, MZ7 cells differ from B16 and Hcmel12 cells in that IP1 production (Fig. 8a), but not ERK1/2 phosphorylation (Fig. 8b), is FR sensitive. Consistently, FR-sensitive inhibition of proliferation was only observed for B16 and Hcmel12 cells (Fig. 8c). We conclude that (i) genetic presence of an activating mutation within Gα_q/11_ (MZ7 harbours Gα_11_R183C, Hcmel12 harbours Gα_q_Q209L) does apparently not suffice to define the molecular subtype of melanoma that responds to Gq inhibition, and (ii) FR effectively suppresses proliferation even in the absence of mutationally activated forms of Gq. We therefore investigated the consequences of Gq inhibition for hallmark behaviours of melanoma cells using the well-established B16 line as model system. FR effectively reduced growth of B16 cells in a concentration-dependent manner, as assessed by trypan blue staining of viable cells (Fig. 8d). Interestingly, in striking contrast to many other anticancer agents, that show direct cytotoxicity, FR did not compromise mitochondrial metabolism (Fig. 8e) suggesting that inhibition of proliferation is achieved without causing cell death. Consistently, flow cytometry analysis indicated that FR-treated cells remained viable and retained their membrane integrity (Fig. 8f). Rather, inhibition of cell growth resulted from cell cycle arrest in the G1 phase (Fig. 8g,h). Notably, no evidence for decomposition of FR was obtained during the assay period (Supplementary Figs 17 and 18), possibly indicating that nature has evolved this peptide to be rather stable.

### FR forces melanoma cells into differentiation

**Figure 9:**
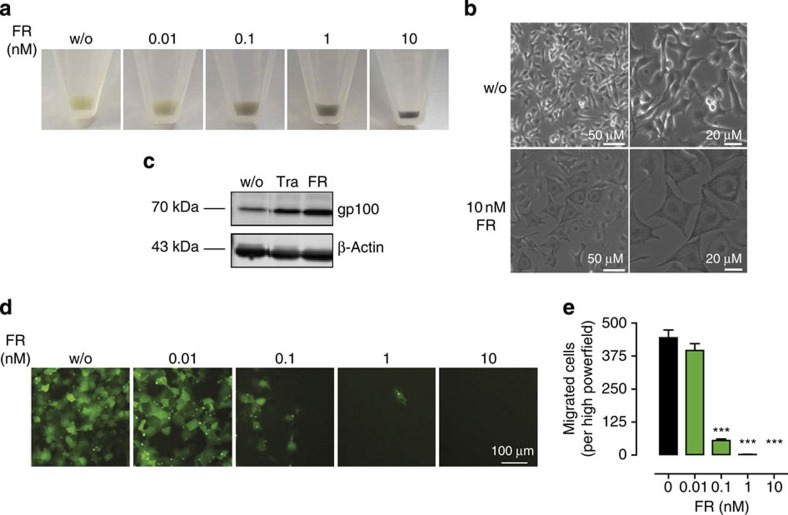
FR forces melanoma cells into differentiation while simultaneously inhibiting migration. (**a**) Phenotype of B16 cell pellet after treatment with various concentrations of FR. (**b**) Light microscopic images of B16 cells cultured without or with FR (10 nM). (**c**) Western blot analysis of the melanoma differentiation marker gp100 in B16 cells without treatment (w/o), after treatement with Tra (Trametinib 1 μM), or after treatment with FR 1 μM. (**d**) Transwell migration of B16 cells after treatment with various concentrations of FR for 24 h. (**e**) Quantification of B16 cell migration shown in **d**. (**e**) *P<0.05, **P<0.01, ***P<0.001 according to a two-tailed Student's *t*-tests. (**a**–**d**) One representative experiment that was repeated twice. (**e**) Means±s.e.m. of *n*=3 experiments performed at least in triplicate.

Inhibition of proliferation via growth arrest and concomitant induction of differentiation has recently been proposed as a novel strategy to target oncogenic signalling in brain tumours^42^. We reasoned that a similar change in cell fate may have occurred in B16 cells when treated with FR, because we noted a gradual decrease in cell pellet volume accompanied by a profound increase in pigmentation (Fig. 9a). Melanosome-containing pigment organelles were also evident in light microscopic images of B16 cells cultured or not in presence of FR (Fig. 9b): FR-treated cells appeared flattened, rather dark, and packed with melanin vesicles, attributes consistent with the adoption of a more differentiated state. Consistently, melanocyte differentiation marker gp100 was upregulated considerably on FR treatment (Fig. 9c). No signs of differentiation were observed in MZ7 cells after treatment with FR in agreement with its lack on proliferation (Supplementary Fig. 19). These results lead us to suggest that Gq inhibition may represent a novel avenue to reprogram melanoma cells to a less aggressive phenotype.

### FR inhibits melanoma cell migration

The migratory features of melanoma cells allow them to spread through lymphatic or blood vessels and form metastases in distant organs. We therefore investigated whether inhibition of Gq signalling would also inhibit melanoma cell migration induced by normal fetal calf serum (FCS)-containing medium. Vehicle-treated melanoma cells displayed a strong migratory capacity in boyden chamber transwell assays, but this behaviour was lost when cells were pretreated with FR (Fig. 9d,e). We infer from these data that Gq signalling is also critically involved in the migration of B16 cells and that inhibition of Gq signalling may represent a novel route to prevent the metastatic spread of melanoma cells with an ‘intrinsic Gq tone'.

## Discussion

Distinct tools and technologies are available today to interrogate the impact of G proteins in GPCR signal transduction by preventing activation of their associated downstream signalling pathways. Among these are minigenes encoding C-terminal Gα peptide sequences^43^, dominant negative forms of Gα subunits^44^, small interfering or short hairpin RNAs^45^, and whole organism or individual cell knockouts^46^. While results obtained with these methods have been very insightful, they cannot replace traditional pharmacological approaches based on target perturbation via chemical inhibition. The latter provide a valuable and complementary view on the biological role of proteins of interest, as they allow a biological readout when proteins are functionally inhibited but physically intact.

PTX^47^ and CTX^48^, two bacterial toxins that mediate their effects via covalent modification of Gα subunits, have been instrumental in defining and diagnosing contribution of Gi and Gs proteins to biological processes^3^,^49^,^50^. Both are available to the research community and have been employed in several thousand publications to address fundamental questions related to G protein function. No such tool with general access for investigators exists for perturbation of Gq proteins. YM254890 (YM), a selective Gq inhibitor^12^, has been withdrawn by Astellas Pharma Inc. and was only available to few research laboratories worldwide. Simplified YM analogues have recently been synthesized but they are not as potent as YM and their selectivity for individual G protein isoforms is unknown^51^,^52^. BIM46187, originally introduced as pan-G protein inhibitor and experimental anticancer agent, is Gq-preferring but not -selective and, unfortunately, changes its preference for G protein inhibition depending on the cellular background in a manner that is unpredictable at present^37^. FR, unlike YM, can be obtained from natural sources that are readily accessible without any restriction^13^. We isolated FR from the leaves of the evergreen plant *A. crenata* and performed extensive *in vitro* investigations to conclude that FR is (i) exceptionally selective for inhibition of Gq family proteins Gq, G11 and G14, (ii) absolutely inert on G protein-independent, arrestin-mediated pathways, (iii) competent to associate with its target Gq via a long-lasting, pseudo-irreversible interaction, (iv) remarkably stable despite its peptide nature, (v) functionally efficacious because it acts as GDI, and (vi) of outstanding value as molecular probe to unravel contribution of Gq signalling in complex biological processes *in vitro* and *ex vivo*.

For the above reasons we argue that FR can clearly be the designated molecule to answer the questions as to what happens when Gq family proteins are inactivated. Biological data on FR are still scarce^53^,^54^,^55^ and its mechanism of action or selectivity for individual G protein isoforms has never been addressed systematically. Our study provides thorough characterization of FR and ‘proof-of-principle' experiments to enable rational application as Gq/11/14-selective inhibitor by the scientific community. ‘Reversibility experiments' led us to suggest that FR might bind to Gq in living cells via long-lasting interaction (Fig. 6). Both in *ex vivo* vessel preparations as well as in intact cells in culture FR effects were remarkably resistant to washout. These results have important implications for development of FR derivatives with altered Gα selectivity profiles because they suggest that characterization of newly developed Gα probes should take into account kinetic properties of inhibitor–target complexes. Indeed, a number of drugs in clinical use gain both selectivity and long duration of action via slow dissociation from their respective targets, a behaviour termed ‘kinetic selectivity'^56^,^57^. Although we cannot deliver a direct estimate of FR dissociation from Gq proteins at present, our data suggest that FR-Gq complexes dissociate slowly. Nevertheless, reversibility experiments, as those exemplified herein (Fig. 6a,c,d) may provide the desired information until suitable radioligands for kinetic profiling will become available.

We were intrigued by the chemical stability of the depsipeptide FR, which we consider extraordinary. Even if incubated over a period of up to 8 days in cell culture medium no decomposition was detected (Supplementary Fig. 18). Peptides usually are metabolically unstable because peptidases attack their amide bonds. However, the cyclic structure of FR confers significant resistance towards their degradation by peptidases. Additional features accounting for resistance of FR to degradation are *N*-methylations of amide bonds as well as unusual amino and hydroxy acid building blocks, which differ greatly from those of normal peptides (Supplementary Fig. 20)^58^,^59^. For the above reasons, FR does not only serve as a pharmacological agent to exclude Gq activity in assays with short incubation times, rather FR can be employed to also investigate contribution of Gq input to cellular processes that need registration over many hours or days, as is the case in cancer drug discovery.

Many potent mitogens exert their proliferative signals via GPCRs that are linked to the Gq family of heterotrimeric G proteins^40^,^41^. The notion that numerous Gq-GPCRs regulate cell proliferation, but also motility and thereby invasive potential of cancer cells led us to posit that inhibition of Gq proteins rather than their linked receptors may be an efficient molecular intervention to target oncogenic signalling at the post-receptor level. We tested this hypothesis in the melanoma B16 cell line and found that selective Gq inhibition was sufficient to attenuate cell proliferation, while simultaneously skewing cellular responses in favour of differentiation. Inhibition of cell growth by FR was due to a G1 cell cycle arrest but not to apoptotic cell death (Fig. 8). This is in contrast to many anticancer agents that also exert anti-growth activity via induction of cell death^60^. The first successful differentiation agent was all-*trans*-retinoic acid to combat acute promyelocytic leukaemia^61^. Recently, forced differentiation of cancer cells has been proposed as a new approach for brain tumour therapy^42^. Our results indicate that FR may hold promise in the search for alternative melanoma therapies because it unifies growth arrest with forced differentiation.

Another serious challenge facing cancer treatment is metastasis, the spread of cancer cells through blood or lymphatic vessels to distant organs. This feature is intimately linked to the cell's capacity to migrate^41^. Migration, however, is not an intrinsic property of cells, but rather a highly regulated and coordinated process orchestrated by numerous extracellular cues, their receptor targets and their associated signalling networks. The capacity of cells to move involves changes in cytoskeletal structure and dynamics with major implication of the G12/13 and Gi-βγ signalling axis^40^. Melanoma B16 cells, however, greatly rely on Gq for migration, and this cellular behaviour is completely abolished by FR. It will be exciting to pinpoint these Gq stimuli in future experiments to compare therapeutic potential of Gq- versus Gq-GPCR inhibition as rational targeted melanoma therapy.

In sum, this is, to the best of our knowledge, the first comprehensive study to reveal the experimental value of FR as tool in probing Gq-sensitive cellular responses with remarkable specificity. It provides framework, knowledgebase and solid foundation for the research community planning to employ FR as reagent in studies of G protein-mediated signal transduction. Just as inhibition of cell responses by pretreatment with PTX has become diagnostic of the involvement of Gi proteins and is applied worldwide by numerous research laboratories, we anticipate FR to stand out at least equally for investigations into the biological relevance of Gq.

## Methods

### Reagents

Cell culture materials were from Invitrogen, U-46619 and nociceptin were from Cayman, GSK-1120212 (Trametinib) were from Selleckchem, and all other reagents were from Sigma Aldrich unless stated otherwise.

### Cell culture

All cell lines were cultivated with 5% CO_2_ at 37 °C in a humidified atmosphere. All media were supplemented with 10% (v/v) FCS, penicillin (100 U ml^−1^) and streptomycin (100 μg ml^−1^).

Native and recombinant HEK293 cells were cultivated in Dulbecco's modified Eagle's medium (DMEM). For CRTH2-HEK293 and GPR55-AD-HEK293, 0.4 mg ml^−1^ geneticin (G418; InvivoGen) was added. ratGPR17-HEK293 and CRTH2-*R*Luc-GFP^2^-β-arrestin2-HEK293 were cultivated with 0.5 mg ml^−1^ G418, and 56 μg ml^−1^ zeocin+0.5 mg ml^−1^ G418, respectively. Media for FFA1-Flp-InT-REx293 and FFA2-Flp-InT-REx293 was supplemented with blasticidin (15 μg ml^−1^)+hygromycin B (100 μg ml^−1^). Expression from the Flp-In locus was induced by doxycycline (1 μg ml^−1^) for 16–18 h.

CHO cells were cultivated in Ham's F12 Nutrient Mix (Ham's F12) with GlutaMAX. For M1-CHO cells, 0.2 mg ml^−1^ G418 was added. FFA4-CHO-Flp-In cells were generated by stably transfecting CHO-Flp-In cells with pcDNA5FRT containing HA-tagged FFA4 and pOG44. For FFA4-CHO-Flp-In selection, hygromycin B (0.4 mg ml^−1^) was added.

The autologous human melanoma cell lines MZ7-MEL (MZ7; exhibits mutations including B-RafV600E and Gα_11_R183C), Skmel28 (also carries the B-RafV600E mutation but is wild type for Gα_q/11_), Mamel15 and Mamel119 (both wild type for B-Raf and Gα_q/11_), Mamel65 (carries a N-Ras Q61K mutation, but is wild type for B-Raf and Gα_q/11_), and the mouse melanoma cell lines Hcmel12 (carries a mutation in Gα_q_Q209L, but is wild type for B-Raf and N-Ras) and B16-eGFP, hereafter B16 (wild type for B-Raf and does not exhibit Gα_q/11_ mutations), were cultured in Roswell Park Memorial Institute (RPMI) 1640 medium (Life Technologies) supplemented with 2 mM L-glutamine, 10 mM non-essential amino acids, 1 mM HEPES (all from Life Technologies), 20 mM 2-mercaptoethanol (Sigma)^62^,^63^. Mutations listed are from the SANGER cancer cell line project database ( http://cancer.sanger.ac.uk/cell_lines) and from inhouse sequencing.

CHO, HEK293, Skmel28 and B16 cells were from the American Type Culture Collection. MZ7 cells were kindly provided by Professor Thomas Hölzel, University of Mainz. Mamel cell lines were kindly provided by Professor Dirk Schadendorf, University of Essen. The Hcmel12 cell line was generated from a 7,12-dimethylbenz(a)anthracene-induced melanoma from HGF-CDK4(R24C) mice^63^.

Melanoma cell lines were routinely authenticated by PCR on characteristic N-Ras and B-Raf mutations. Hcmel12 cells were routinely authenticated by PCR on CDK4. All cell lines were tested for mycoplasma contamination by PCR on a regular basis and were found to be mycoplasma-free.

*In vitro* morphology was assessed using a TE Eclipse microscope (Nikon).

For inhibitor experiments or pan-G protein activation, cells were pretreated overnight (50 ng ml^−1^ PTX; 100–200 ng ml^−1^ CTX) or for 60–120 min (300 μM AlF_4_^−^; 1–10 μM FR) unless stated otherwise.

### Site-directed mutagenesis

Mutations of the mouse Gα_q_ (Gα_q_R183C and Gα_q_Q209L) cDNA, HA-tagged in pcDNA3.1(+), were generated by quick change site-directed PCR mutagenesis^64^ with the following primer (5′–3′):

Gα_q_Q209L fwd: 5′-GATGTAGGGGGCCTAAGGTCAGAGAG-3′

Gα_q_Q209L rev: 5′-CTCTCTGACCTTAGGCCCCCTACATC-3′

Gα_q_R183C fwd: 5′-CAACAAGATGTGCTTAGAGTTTGTGTCCCCACCACAGGGATCATC-3′

Gα_q_R183C rev: 5′-GATGATCCCTGTGGTGGGGACACAAACTCTAAGCACATCTTGTTG-3′

Successful mutations were verified by DNA sequencing.

### Generation of Gα_q/11_ knockout HEK293 cells

Gα_q_ and Gα_11_, which are encoded by the *GNAQ* and the *GNA11* genes, respectively, were simultaneously targeted by a CRISPR-Cas9 system^65^ with some modifications. *GNAQ*-targeting sgRNA sequence (5′-AAACAAGAAAGATCTTCTAGA-3′; a part of Xba I-recognizing sequences (TCTAGA) is underlined) was inserted into the Bbs I site of the pX330-U6-Chimeric_BB-CBh-hSpCas9 vector (pX330; a gift from Feng Zhang, Broad Institute; Addgene plasmid # 42230) using two synthesized oligonucleotides (5′-CACCGAAACAAGAAAGATCTTCTAG-3′ and 5′-AAACCTAGAAGATCTTTCTTGTTTC-3′; FASMAC, Japan). Similarly, *GNA11*-targeting sgRNA sequence (5′-AGGGTACTCGATGATGCCGG-3′; complementary to the gene transcription direction; Hap II-recognizing sequences is underlined) was inserted using a set of oligonucleotides (5′-CACCGAGGGTACTCGATGATGCCGG-3′ and 5′-AAACCCGGCATCATCGAGTACCCTC-3′). Correctly inserted sgRNA sequences were verified by a Sanger sequencing method (FASMAC, Japan). HEK293A cells were seeded in a 12-well culture plate at a density of 200,000 cells per well in DMEM 2 (Nissui Pharmaceutical, Japan) supplemented with 10% fetal bovine serum (Gibco, Thermo Fisher Scientific) and penicillin/streptomycin and transfected with a mixture of the *GNAQ*-targeting vector (0.25 μg), the *GNA11-*targeting vector (0.25 μg) and a pGreen Lantern vector encoding a green fluorescent protein (0.1 μg) using a Lipofectamine 2000 Reagent (Life Technologies, CA, USA). Twenty-four hours post transfection, the cells were detached and GFP-positive cells (∼30% of injected cells) were isolated using a cell sorter (SH800, Sony, Japan). The GFP-positive cells were diluted with culture media, seeded in a 96-well plate and incubated for ∼2 weeks with addition of fresh media every 5 days. Wells were routinely checked for their colony appearance and wells containing an apparent single colony were selected for passages. A half of the detached cells was analysed for mutations in the *GNAQ* and the *GNA11* genes using PCR and restriction enzyme digestion. The following combination of PCR primers and a restriction enzyme was used to screen mutations: for the *GNAQ* target, 5′-CCGAATGGAGGAAAGCAAGG-3′, 5′-ATCAGTTTCAACACGCAGGC-3′ and Xba I (TAKARA BIO, Japan); for the *GNA11*, and 5′-TGTTGCAGCTACCTGACCG-3′, 5′-GAGCCTCTCAGTGCCCCCTC-3′ and Hap II (TAKARA BIO, Japan). PCR condition was 95 °C for 2 min, followed by 35 cycles of 95 °C for 15 s, 64 °C for 30 s and 72 °C for 30 s. PCR product (5 μl) was mixed with 0.5 μl of a corresponding restriction enzyme in a reaction volume of 10 μl at 37 °C for 1 h. The digested solution was applied on 3% agarose gel containing ethidium bromide and separated by electrophoresis and DNA fragments were visualized under ultraviolet light. Candidate clones that contained restriction enzyme-resistant PCR fragments were assessed for functional analysis of Gα_q/11_ using a TGFα shedding assay^66^ as well as Ca^2+^ response using a FLIPR Calcium 5 Assay Kit (Molecular Devices).

### Isolation and purification of FR900359

Dried leaves of *A. crenata* were extracted three times with methanol (MeOH). The extract was fractionated by vacuum liquid chromatography over RP_18_ material using gradient elution from MeOH:H_2_O (3/7) to 100% MeOH. The MeOH fraction was subsequently separated on Sephadex LH-20 with MeOH as eluent. Reversed phase HPLC separation of the FR containing fraction (column: YMC C_18_ Hydrosphere, 250 × 4.6 mm, 3 μm; MeOH:H_2_O (8:2), 0.7 ml min^−1^) yielded pure FR (retention time 24 min). The previous commercial name for FR was UBO-QIC.

### Stability of FR900359

HPLC was performed on a Waters HPLC system equipped with an in-line degasser AF, a 600 controller, a 717 plus autosampler, and a 996 photodiode array detector. The analyses were done using an isocratic method (80/20 MeOH/H_2_O; flow: 0.7 ml min^−1^) with an YMC Hydrosphere RP_18_ column (250 × 4.6 mm, 3 μm).

To analyse the stability of 100 μM FR in Ham's F12, the concentration was analysed at day 0, 3 and 8, relative to 100 μM FR in methanol. Ca^2+^ mobilization assays were used to determine the stability in cell-based assays.

### LC/electrospray ionisation MS measurements

Mass spectra were recorded on an API 2000 liquid chromatography-tandem mass spectrometry (LC-MS)/MS spectrometer (electron spray ion source, Applied Biosystems) coupled with an Agilent 1100 HPLC system using a Phenomenex Luna HPLC C18 column (50 × 2.00 mm, particle size 3 μm). Compound purity was determined by HPLC-ultraviolet using the following procedure. A amount of 10 μl of compound (1 mg ml^−1^ in methanol) was injected and eluted with a gradient of water/methanol containing 2 mM ammonium acetate from 60:40 to 0:100 for 10 min, and subsequently with 0:100 for 10 min at a flow rate of 300 μl min^−1^, starting the gradient after 10 min. Ultraviolet absorption was detected from 220 to 400 nm using a diode array detector.

### Synthesis of FR900359-*red*

In all, 10% Pd/C (0.8 mg) was added to 1.00 mg (0.998 μmol) FR in MeOH (1 ml) and the reaction mixture was stirred under a H_2_ atmosphere (17 psi) for 9 h. After hydrogenation of the starting material was completed (monitored by LC-MS) the mixture was filtered (syringe filter, 0.45 μm, or a thin pad of celite). The filter was washed with methanol and the filtrate was evaporated. The residue was dried in vacuum (10^−2^ mbar) to obtain ‘FR-*red*' (PSB-13900) (0.85 mg, 0.846 μmol; 85%).

### IP1 and cAMP accumulation assay

Quantification of intracellular cAMP and IP1 was performed using the HTRF-cAMP dynamic kit and the HTRF-IP1 kit, respectively, on a Mithras LB 940 reader (Berthold Technologies) according to the manufacturer's instructions^37^. For cAMP assays with M2-CHO cells, the Gi-biased muscarinic agonist iper-6-phth (ref. 30) was applied.

For washout experiments, cells were pre-incubated for 1 h with FR (1 μM) and then washed three times for 5 min with 750 μl PBS, resuspended in fresh stimulation buffer, and seeded into a 384-well plate. For IP1 experiments with Gα_14_ and Gα_16_ (UMR cDNA resource center), HEK293 cells were transiently co-transfected with ORL1 cDNA using FuGENE HD transfection reagent (Promega).

### Calcium mobilization

Intracellular Ca^2+^ mobilization was measured using the calcium 5 assay kit (Molecular devices). Briefly, ratGPR17-HEK293 cells (60,000 per well) were seeded (24 h before the assay) into poly-D-lysine (PDL)-coated black 96-well culture plates with clear bottom. Cells were loaded with calcium 5 indicator dye for 60 min, stimulated with MDL29,951 and intracellular calcium mobilization was detected with the FlexStation 3 Benchtop Multimode Plate Reader.

Stability of FR was tested by either storing FR in cell media or adding FR to the cell culture flask. Samples were taken for up to 8 days.

### Western blot detection of ERK1/2 phosphorylation

In all, 50,000 FFA4-CHO-Flp-In cells per well were seeded in 6-well plates and incubated overnight at 37 °C. Cells were washed and incubated in serum-free DMEM for 2 h before addition of vehicle or TUG891 (10 μM final) for 5 min. Media were replaced by Tris-buffered saline (TBS) containing 1% NP-40, 0.5% sodium deoxycholate, phosphatase and protease inhibitors (Complete, Roche Diagnostics). Lysates were cleared by centrifugation at 20,000*g* for 20 min. A amount of 2 μg protein (of the supernatant) of each sample was separated by SDS–PAGE on 10% gels that were transferred to polyvinylidene difluoride, blocked and probed with antibodies for pERK1/2 (#9101) and total ERK1/2 (#9102) at 1:1,000 dilution (Cell Signaling Technology). Blots were scanned and bands were quantified using Alphaimager software (Alpha Innotech).

### HTRF-based ERK1/2 phosphorylation assay

Quantification of phosphorylated ERK1/2 levels was performed using the phospho-ERK (Thr202/Tyr204) cellular assay kit (Cisbio). Melanoma cells were seeded into 96-well plates at a density of 25,000 or 50,000 cells per well and cultured overnight. The next day, medium was aspirated and cells were treated with 1 μM FR, 1 μM Trametinib, 1 μM Vemurafenib, or medium for 1 h at 37 °C. Subsequently, medium and inhibitors were removed and 50 μl lysis buffer were added to each well. The plates were incubated for 30 min at room temperature with shaking to lyse the cells and then frozen overnight at −20 °C. Lysates (16 μl) were transferred to a white 384-well plate. Anti-phospho-ERK1/2-d2 (2 μl) and anti-phospho-ERK1/2-Eu3^+^-cryptate (2 μl) were added to each well and plates were incubated in the dark for 2 h at room temperature. Time-resolved fluorescence resonance energy transfer (FRET) signals were measured using the Mithras LB 940 multimode reader (Berthold Technologies).

### Melanoma immunoblots

Melanoma cells were seeded into 6-well plates in complete RPMI medium. Six hours after seeding cells were treated with 1 μM FR, 1 μM Trametinib, or medium for 72 h at 37 °C to analyse gp100 and microphthalmia-associated transcription factor (MITF) expression. Melanoma cells were directly lysed in Laemmli buffer and subsequent incubation for 5 min at 95 °C. Total cell lysates were separated using 10% SDS–PAGE and proteins were transferred to a nitrocellulose membrane (GE Healtcare) by wet blotting (BioRad Mini-PROTEAN Tetra handcast system). After 1 h of blocking with 5% bovine serum albumin (GE Healtcare) in TBS with 0.5% Tween-20, membranes were incubated with primary antibodies overnight at 4 °C. Corresponding secondary antibodies and the Odyssey Sa Imaging system (LI-COR Biosciences) were used for protein detection. Primary antibodies: β-Actin (C4, Santa Cruz sc-47778) at 1:1,000 dilution, gp100 (abcam ab-52058) at 1:500 dilution and microphthalmia-associated transcription factor (MITF) (abcam ab-24875) at 1:1,000 dilution, secondary antibodies (all provided by LI-COR Biosciences): donkey anti-mouse IRDye 680 LT, donkey anti-goat IRDye 800 CW.

### Label-free cellular-based assays

DMR was recorded with the Corning Epic biosensor^30^,^31^ or with the PerkinElmer Ensight with the following modifications: for HEK293, CRTH2-HEK293 and FFA1-HEK293, 15,000, 18,000 and 18,000 cells per well, respectively, were seeded 18–24 h before experiment. For GPR55-HEK293 cells, 12,000 cells per well were seeded 48 h before experiment and after 24 h media was replaced by assay buffer for starvation conditions.

Bioimpedance (CellKey) assays were performed^67^ with the following modifications: 11,000 (3,000 for CRTH2-HEK293 and FFA1-Flp-InT-REx293) cells per well were seeded on PDL-coated 384-well biosensor plates for 18–24 h to grow to confluence. GPR55-HEK293 cells were seeded 48 h before the assay and after 24 h medium was replaced by assay buffer (Hank's Balanced Salt Solution (HBSS) with 20 mM HEPES).

### Bioluminescence resonance energy transfer

Receptor and G protein constructs were transiently co-transfected into HEK293 or HEK293T cells using polyethylenimine (Polysciences Inc.). Generation of G protein BRET probes was described previously in detail^17^,^18^ (see references within). Forty-eight hours after transfection, cells were washed and resuspended in PBS with 0.1% (w/v) glucose at room temperature. Cells were pretreated with 100 nM FR or vehicle for 30 min and then distributed (70–80 μg of protein per well) in a 96-well microplate (Wallac, PerkinElmer Life and Analytical Sciences) and incubated in the presence of increasing doses of ligands for 2 min. BRET^2^ between *R*Luc8 and GFP^10^ or between *R*LucII and GFP^10^ was measured after the addition of the *R*Luc substrate coelenterazine 400a (5 μM, Interchim). BRET^2^ readings were collected using a modified Infinite F500 (Tecan Group Ltd), a Mithras LB 940 multimode reader (Berthold Technologies), or a Synergy2 (BioTek) microplate reader. The BRET^2^ signal was calculated as ratio between GFP^10^ emission and the light emitted by *R*luc8 or *R*lucII, respectively. The changes in BRET induced by the ligands were expressed on graphs as ‘ligand-promoted BRET' using the formula: ‘BRET ratio with ligand—BRET ratio without ligand'.

To estimate β-arrestin2 recruitment to FFA4, plasmids encoding FFA4 fused to enhanced yellow fluorescent protein at its C-terminus and β-arrestin2 fused to Renilla luciferase were co-transfected into HEK293 cells. Twenty-four hours post transfection, cells were distributed into white 96-well plates and then maintained in culture for another 24 h before their use. To conduct the assay, cells were first washed in HBSS before addition of Renilla luciferase substrate coelenterazine h (5 μM) and the ligand of interest. Cells were incubated at 37 °C for 30 min before luminescence at 535 and 475 nm was recorded using a Pherastar FS. The ratio of luminescence at 535/475 nm was then used to calculate the BRET response.

For BRET^2^ assays on HEK293 cells stably expressing CRTH2-Luc and GFP^2^-β-arrestin2, cells were detached and resuspendend in HBSS with 20 mM HEPES at a density of 1.06 × 10^6^ cells per ml. Cell suspension (170 μl) was distributed into white 96-well microplates and incubated in the presence of 10 μl buffer or agonist for 5 min before substrate addition. DeepBlueC coelenterazine (Gold Biotechnology, MO, USA; 20 μl per well) was injected by injector 3 to yield a final concentration of 5 μM. To detect BRET, light emissions at 400 and 515 nm were measured sequentially using a Mithras LB 940 instrument. The BRET signal (BRET ratio) was determined by calculating the ratio of the light emitted by the fluorescence acceptor (515 nm) and the light emitted by Rluc (400 nm). The cells were pretreated with PTX for 16–20 h and FR for 30 min.

### Radioligand binding

For whole-cell binding assays^39^, M1-CHO cells were collected and 70,000 cells per well were incubated with 0.2 nM [^3^H]*N*-methylscopolamine ([^3^H]NMS; PerkinElmer Inc.) and different concentrations of non-labelled competitor with or without 1 μM FR in assay buffer (HBSS supplemented with 20 mM HEPES; pH 7.0) in a 96-well microtiter plate (Fischer Scientific GmbH) at 28 °C in a final volume of 300 μl for 2 h. For radioligand binding experiments performed with CHO-M1 mebranes^39^,^68^, membranes (20–40 μg ml^−1^) were incubated with 0.2 nM [^3^H]NMS and different concentrations of non-labelled competitor with or without 1 μM FR in a HEPES buffer (10 mM HEPES, 10 mM MgCl_2_, 100 mM NaCl, pH 7.4) at 30 °C in a final volume of 300 μl for 2 h in a 96-well microtiter plate.

Binding experiments were terminated by rapid vacuum filtration through a glass fibre filter (PerkinElmer Inc.) and filter-bound radioactivity was determined by solid scintillation counting. Non-specific binding was determined in presence of 10 μM atropine.

### Gα_q_ purification

Gα_q_Δ34 (containing the first 28 residues of rat Gα_i1_ fused to mouse Gα_q_ lacking the first 34 residues, and with a TEV-cleavage site between the Gα_i1_ and Gα_q_ sequences) was purified after expression from a pFastBac1 vector in insect cells^37^. Purified Gα_q_Δ34 (∼7 mg ml^−1^) was stored at −80 °C in 20 mM HEPES pH 8, 0.2 mM dithiothreitol, 2% (v/v) glycerol, 100 mM NaCl, 0.1 mg ml^−1^ PMSF augmented with 10 mM NaF, 30 μM AlCl_3_, and 5 mM MgCl_2_.

### GDP exchange assay

[^3^H]GDP exchange by Gα_q_ was measured based on previous protocols^12^,^37^. Briefly, purified Gα_q_Δ34 (100 nM) was incubated with 1 μM [^3^H]GDP (42,500 c.p.m. pmol^−1^) and 50 mM (NH_4_)_2_SO_4_ for 18 h at 20 °C in assay buffer A (50 mM HEPES pH 7.5, 1 mM EDTA, 0.9 mM MgSO_4_, 1 mM dithiothreitol, and 0.05% Genapol C-100). Excess [^3^H]GDP was removed from Gα_q_ using a ZebaTM spin desalting column following the manufacturer's protocol (Thermo Scientific). Approximately 20% of Gα_q_ incorporated the radiolabelled nucleotide. The reaction mixtures were then mixed with the same volume of assay buffer A in the presence of 0.1–2% dimethylsulphoxide (DMSO), 0–200 nM FR and 1.5 M (NH_4_)_2_SO_4_ to reach final concentrations of 0.5–1% DMSO, 0–100 nM FR and 750 mM (NH_4_)_2_SO_4_ in the reaction. Dissociation of [^3^H]GDP was monitored at 20 °C by stopping the reaction by addition of 4 ml of an ice-cold wash buffer (20 mM Tris pH 8.0, 100 mM NaCl and 2 mM MgSO_4_). The quenched mixtures were filtered through nitrocellulose membranes, the membranes were washed four times with ice-cold wash buffer, and the filters air-dried for at least 10 min. Radioactivity was quantified by scintillation spectrometry.

### Nucleotide dissociation

Nucleotide dissociation assays were performed with AtGpa1 (pPROEXHTb-AtGpa1ΔN36) that was expressed in and purified from BL21(DE3)PLysS *Escherichia coli*. Assays were carried out in a final volume of 1.5 ml PBS (25 mM potassium phosphate, 100 mM KCl, pH 7.0) containing 200 nM 2′-O-(*N*-methylanthraniloyl)-guanosine 5′-diphosphate (MANT-GDP) in a quartz cuvette^15^. Briefly, purified AtGpa1 was equilibrated with either DMSO or FR in DMSO for 20 min at room temperature. Equilibrated protein mix was added to the cuvette containing MANT-GDP and observed for full loading of fluorescent nucleotide. To initiate MANT-GDP dissociation, 10 μM unlabelled GDP was added and the loss of FRET emission quantified.

### Fast quantitative cysteine reactivity

Purified AtGpa1 (1.8 μM final) was pre-equilibrated with either DMSO or FR (10 μM final) for 20 min at room temperature before dilution in cold PBS (pH 7.0) with 50 μM GDP and chilled on ice. 4-(aminosulfonyl-7-fluoro-2,1,3-benzoxadiazole (ABD; TCI America; A5597) was added (1 mM) to the chilled protein mix and distributed to pre-chilled PCR strip tubes. Unfolding was carried out in a gradient thermocycler (Biometra TProfessional Thermocycler) for 3 min at indicated temperatures from 27 to 67 °C, transferred back to ice, and ABD labelling quenched by addition of 0.1 N HCl (ref. 69). Samples were transferred to a 384-well plate and ABD fluorescence measured in a BMG Labtech PHERAstar plate reader (excitation: 400 nm; emission: 500 nm).

### Isometric force measurements

Mouse tail artery was dissected and cut into 2-mm-long rings in cold (4 °C) low-calcium physiological saline solution containing 118 mM NaCl, 5 mM KCl, 1.2 mM MgCl_2_, 1.5 mM NaH_2_PO_4_, 0.16 mM CaCl_2_, 10 mM glucose and 24 mM HEPES, pH 7.4. Arterial rings were mounted on a small vessel wire myograph (Multi Myograph 610 M, Danish Myo Technology). A computer-assisted normalization protocol was exerted to pre-stretch vascular rings to 0.9 × L100 with L100=diameter mimicking a transmural pressure of 100 mm Hg. Rings were equilibrated for 20 min in physiological saline solution: 118 mM NaCl, 5 mM KCl, 1.2 mM MgCl_2_, 1.5 mM NaH_2_PO_4_, 1.6 mM CaCl_2_, 10 mM glucose and 24 mM HEPES, pH 7.4. The solution was bubbled with 100% oxygen and heated to 37 °C. Before the experiments, tail arteries were maximally contracted with PE (10 μM).

### Cell proliferation assays

For analysis of the proliferation of human and mouse melanoma cells, cells were seeded into 6-well plates (1 × 10^5^ per well) in complete RPMI medium. Six hours later FR or GSK-1120212 (Trametinib) was added in indicated concentrations. Control groups were left untreated. After 72 h cells were collected and viable cells were counted using trypan blue.

For analysis of growth properties of HEK293 cells, 10^5^ HEK 293 cells per well were seeded on a 6-well tissue culture plate. After 3 h cells were treated with either 10 μmol FR or 0.1% DMSO. After 72 h cell number was determined by counting in a Neubauer chamber.

### Cell viability assays

B16 cell viability in the presence of various concentrations of FR was measured using the XTT-based cell proliferation kit II (Fa. Roche) according to the manufacturer's protocols^37^. Viability of HEK293 cell lines was assessed using a fluorimetric detection of resorufin (CellTiter-Blue Cell Viability Assay, Promega). Specifically, Gα_q/11_ ko-HEK293 and HEK293 cells were seeded at a density of 20,000 and 25,000 cells per well into black 96-well poly-D-lysine-coated plates with clear bottom, respectively. Three hours after seeding cells were treated with 0.3% DMSO or FR dissolved in medium for 24 h. To detect cell viability, CellTiter-Blue reagent was added and cells were incubated for 1 h at 37 °C according to the manufacturer's instructions. Fluorescence (excitation 560 nm, emission 590 nm) was measured using a FlexStation 3 Benchtop Multimode Plate Reader and data were expressed as percentage of cell viability relative to DMSO control.

### Flow cytometry

B16 cells were seeded into 6-well plates (1 × 10^5^ per well) in complete RPMI medium. 6 h later FR was added in various concentrations. Control groups were left untreated. After 72 h cells were collected and apoptosis induction and cell cycle arrest was analysed using standard protocols with annexin V (BD PharMingen) and propidium iodide. Data were acquired with a FACSCanto flow cytometer (BD Biosciences) and analysed with FlowJo software (TreeStar, V7.6.5 for Windows).

For apoptosis analysis of HEK293 cells, cells were stained for Annexin V by use of the Annexin V-FITC Apoptosis Detection Kit (Calbiochem) according to the manufacturer's instructions.

### Immunofluorescence stainings of HEK cells

Cells grown on coverslips were fixed with 4% paraformaldehyde in PBS and stained with antibodies as follows (in 0.2% Triton X in PBS, supplemented with 5% donkey serum; 2 h at room temperature): Ki67 MIB-1 (1:200, Covance) and vimentin (1:1,000, Chemicon). Primary antibodies were visualized by secondary antibodies conjugated to Cy5 (1:400, Jackson ImmunoResearch) diluted in 1 μg ml^−1^ Hoechst 33342 (nuclei staining) at room temperature for 1 h. For documentation an inverted fluorescence microscope (Axiovert 200; Carl Zeiss MicroImaging, Inc.) equipped with a slider module (ApoTome; Carl Zeiss MicroImaging, Inc.), using filters for DAPI, GFP, Cy3 and Cy5, x25, x40 DIC Plan Apochromat oil objectives, an ebx 75 light source and an AxioCam MRm digital camera was used. Pictures were generated with the Axiovision Rel. 4.8 software (Zeiss).

### Transwell migration assay

Migration of B16-eGFP cells was quantified using transwell assays. Briefly, 5 × 10^4^ melanoma cells in 250 μl RPMI 1640 with 1% FCS were placed in the upper chamber of uncoated polyethylene terephthalate (PET) filters (BD Biosciences, 8 μm pore size) in a 24-well plate and subsequently incubated at 37 °C/5% CO_2_ to adhere. Six hours later, FR was added in various concentrations. RPMI containing 1% FCS was added to the lower chamber. Twenty-four hours later, cells in the upper chamber were removed by cotton swab. Transmigrated cells on the lower surface of the membrane were counted in three high-power fields (magnification × 100) using a TE Eclipse microscope (Nikon).

### Modelling

For docking studies we used a YM254890-based structure derived from pdb 3ah8 (ref. 12). Therefore, the respective residues of YM were modified and geometry optimized using a semi-empirical quantum mechanics approach that is implemented in Yasara's YAPAC module (Yasara ‘structure' 14.5.1). The target protein structure was also derived from the crystal structure of the Gα_q_βγ-YM complex^12^ (pdb 3ah8). Docking was performed on the heterotrimeric Gα_q_/βγ complex using autodock VINA default parameters. The set-up was done with the YASARA molecular modelling program, and the best scoring result of 24 docking runs was subjected to further analysis. To guide the docking runs, the docking cell was placed around the YM binding epitope revealed by Nishimura *et al.*^12^ Ligands and receptor residues were kept flexible during the docking runs. Structural alignments were performed with the MUSTANG or Theseus algorithm. Molecular dynamic simulations were performed with the YASARA molecular modelling program (Yasara ‘structure' 14.5.1). Hydrogen bond analysis was performed after energy minimization of the inhibitor-protein complex in explicit water, using the particle-mesh Ewald (PME) method to describe long-range electrostatics at a cutoff distance of 7.8 Å and the Yasara2 force field at physiological conditions (0.9% NaCl, 298 K, pH 7.4).

### MD simulations

The structures (Gα_q_-FR and Gα_q_ only) were equilibrated for about 80 ns in explicit water using particle-mesh Ewald (PME) method to describe long-range electrostatics at a cutoff distance of 7.86 Å and the AMBER03 force field at physiological conditions (0.9% NaCl, 298 K, pH 7.4) followed by a 40 ns production run. Simulation time step interval was chosen as 2.5 fs. Supplementary Fig. 21 illustrates that the timescale of our simulations is sufficient as illustrated by the RMSD time traces. Molecular graphics were created with YASARA ( www.yasara.org) and POVRay ( www.povray.org).

### Data analysis

All data were analysed using GraphPad Prism Version 5.00, 5.01 or 6.05 (Graphpad Software, Inc). Data points from concentration-effect relationships of individual functional experiments or radioligand binding studies were fitted to a four parameter logistic function (equation (1)) with a subsequent extra-sum-of squares *F*-test to decide whether the steepness of the curve differed from −1 (*P*<0.05).


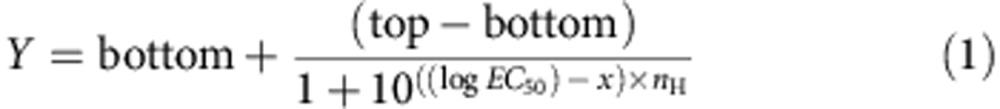


For radioligand displacement studies with [^3^H]NMS or [^3^H]iperoxo performed in membranes of CHO-M1 cells, an extra-sum-of-squares *F*-test was conducted to decide whether the data points were best described using a one site competition (equation (2)) or a two site competition (equation (3); *F*-test, *P*<0.05).


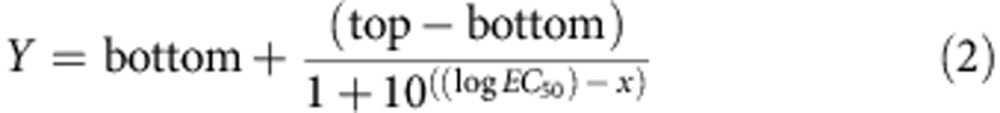



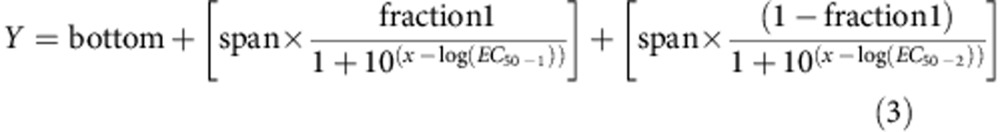


All data presented are mean values±s.e.m. of n independent experiments, unless stated otherwise. Comparison between two experimental groups was based on a two-tailed Student's *t*-test. *P* values were considered as significant (*) if *P*<0.05, as very significant (**) if *P*<0.01 and as extremely significant (***) if *P*<0.001.

## Additional information

**How to cite this article:** Schrage, R. *et al.* The experimental power of FR900359 to study Gq-regulated biological processes. *Nat. Commun.* 6:10156 doi: 10.1038/ncomms10156 (2015).

## Supplementary Material

Supplementary Information Supplementary Figures 1-21, Supplementary Tables 1-2 and Supplementary References.
