# Supplementary material for: The experimental power of FR900359 to study Gq-regulated biological processes
Source: Nat Commun. 2015 Dec 14;6:10156. doi: 10.1038/ncomms10156 (PMC4682109; doi:10.1038/ncomms10156)
Supplement: Supplementary Information — Supplementary Figures 1-21, Supplementary Tables 1-2 and Supplementary References. [file ncomms10156-s1.pdf]

# Supplementary Figure 1

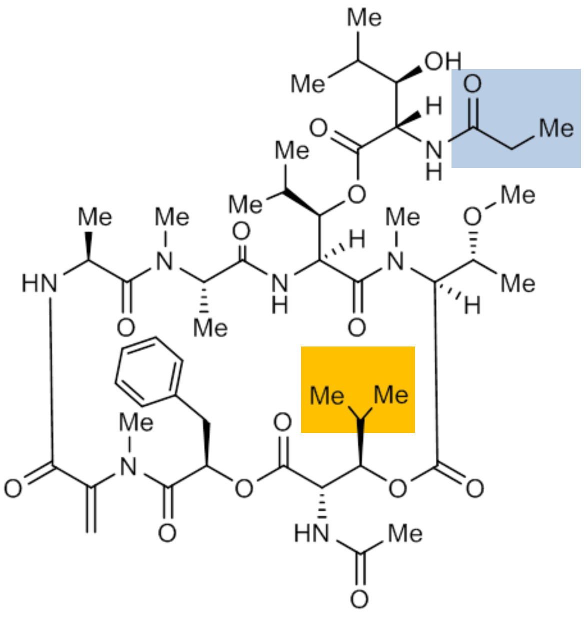

FR900359

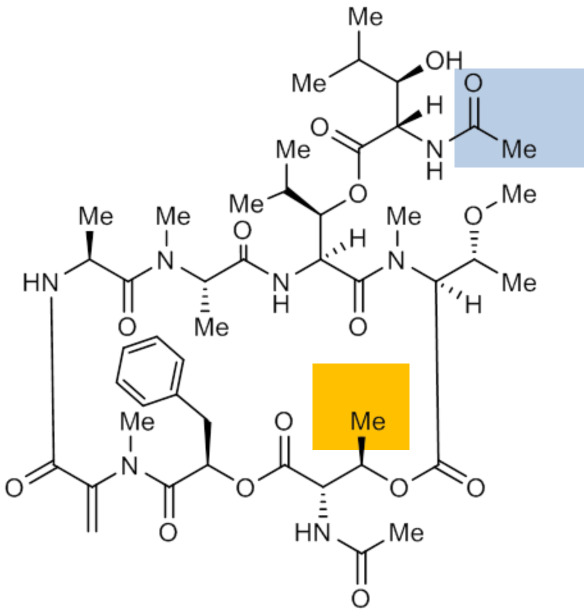

YM254890

**Supplementary Figure 1: Comparison of the structures of FR900359 (FR) and YM254890 (YM).** In FR a methyl group of YM is replaced by an isopropyl moiety (yellow box) and the acetyl group attached to hydroxy-leucine in the side chain of YM is substituted by a propionyl residue (blue box).

# Supplementary Figure 2

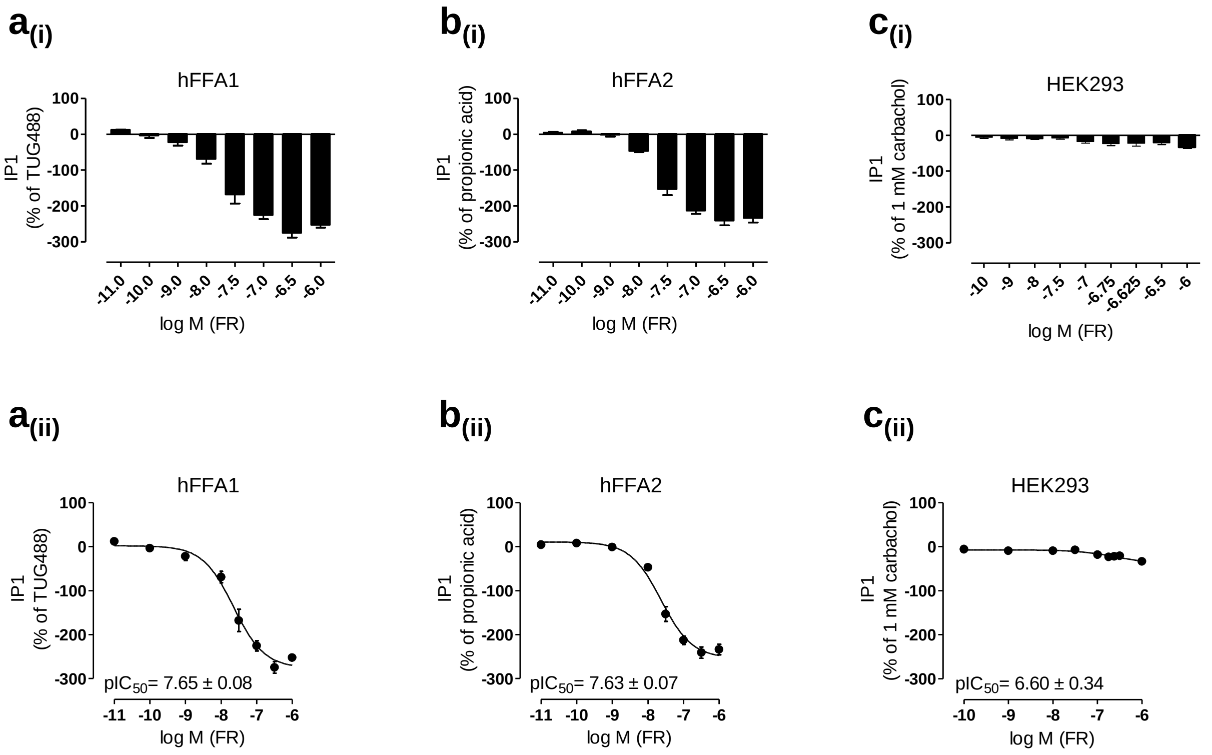


**Supplementary Figure 2: Influence of FR on cell-intrinsic inositolphosphate (IP1) production.** (**a-c**) FFA1-, FFA2-HEK, and native HEK293 cells were pretreated for 1 h with increasing concentrations of FR and intrinsic IP1 accumulation was quantified. Depression of basal IP1 production is particularly apparent in FFA1-HEK ($\mathbf{a}_{(i)}$) and FFA2-HEK cells ($\mathbf{b}_{(i)}$), which harbor constitutively active free fatty acid receptors. IP1 accumulation is hardly diminished in the native host cell line ($\mathbf{c}_{(i)}$). ($\mathbf{a}_{(ii)}$-$\mathbf{c}_{(ii)}$) Quantification of bar diagrams as concentration effect relationships. Data shown are means + SEM ($\mathbf{a}_{(i)}$-$\mathbf{c}_{(i)}$) or means ± SEM ($\mathbf{a}_{(ii)}$-$\mathbf{c}_{(ii)}$) of at least three independent experiments, each conducted in triplicate.

# Supplementary Figure 3

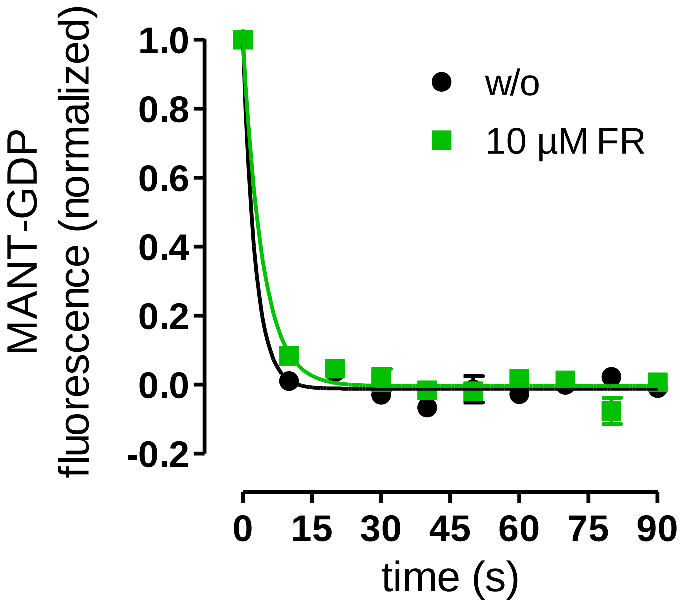


**Supplementary Figure 3: FR does not interfere with AtGpa1 nucleotide exchange.** Measurement of the effect of FR on nucleotide exchange of plant AtGpa1 (Arabidopsis thaliana Gpa1) using the fluorescent GDP analog MANT-GDP. Data are mean ± SEM of three independent experiments and curve fit was based on a one-phase exponential decay. w/o, without FR.

## Supplementary Figure 4

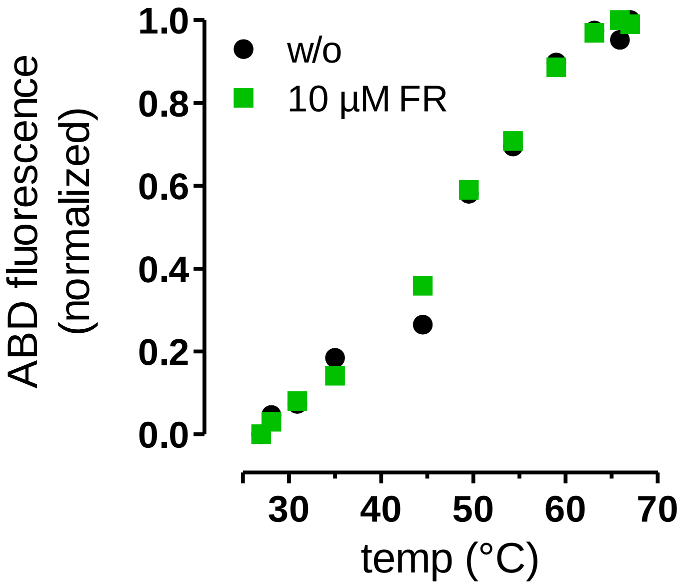


**Supplementary Figure 4: FR does not impair the stability of plant AtGpa1.** Analysis of AtGpa1 protein thermal stability in absence or presence of 10 µM FR measured by fast quantitative cysteine reactivity (fQCR). Shown are data of a representative experiment. ABD, p-aminobenzamidine. w/o, without FR.

# Supplementary Figure 5

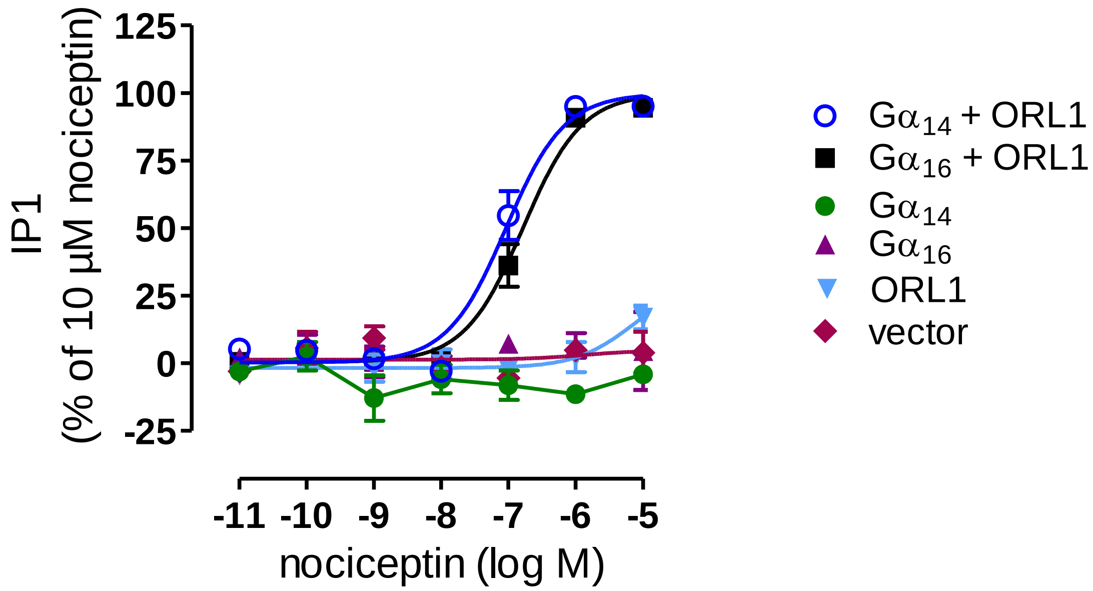


**Supplementary Figure 5: Gi-sensitive ORL1 receptor requires G$\alpha_{14}$ or G$\alpha_{16}$ for productive interaction with PLCβ.** HEK293 cells transfected with the indicated plasmid cDNAs were treated with the ORL1 agonist nociceptin and IP1 production was determined. Cells transfected with empty vector, ORL1 or Gα subunits alone did not respond to ORL1 agonist nociceptin. Shown are mean values ± SEM of 2-4 independent experiments, each performed in duplicate.

# Supplementary Figure 6

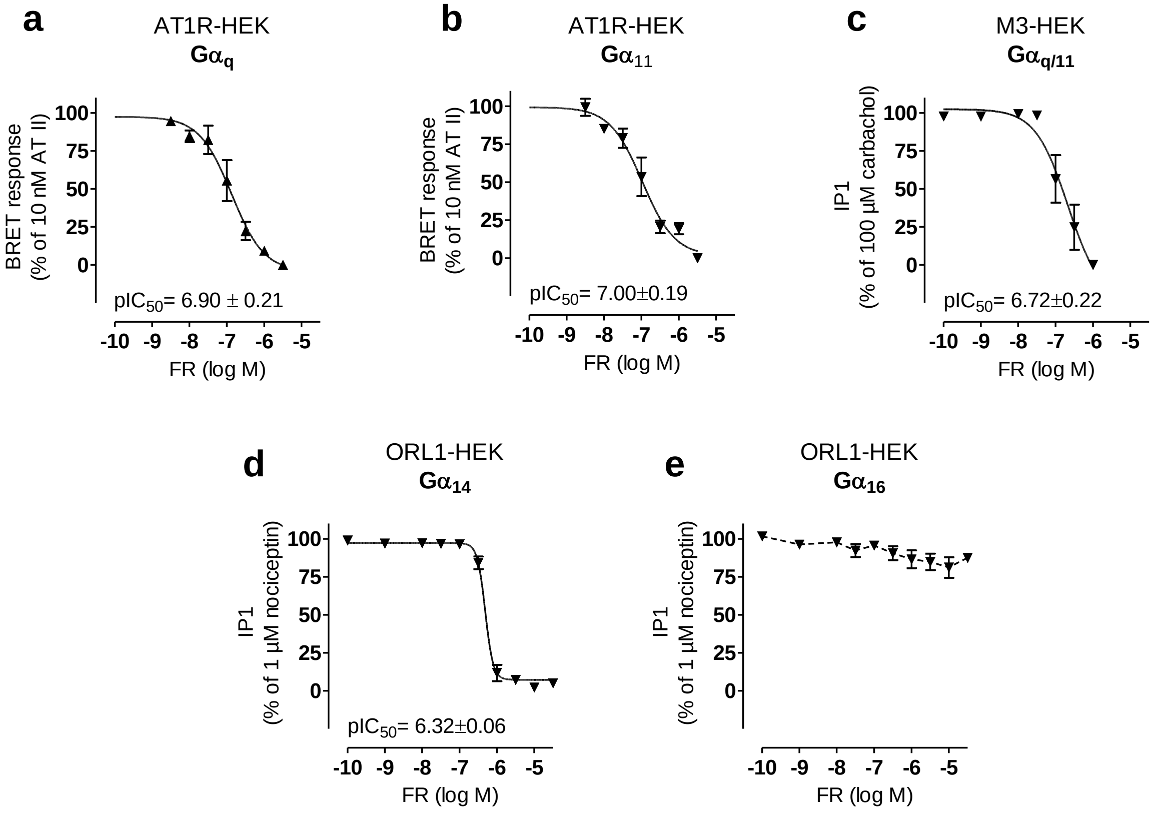


**Supplementary Figure 6: FR inhibits Gα$_q$, Gα$_{11}$, and Gα$_{14}$ with similar potency, but does not block activation of Gα$_{16}$.** (**a,b**) HEK293T cells transfected to co-express (**a**) Gα$_q$-*R*Luc8, or (**b**) Gα$_{11}$-*R*Luc8, along with GFP$^{10}$-Gγ$_2$, unlabeled Gβ$_1$, and the angiotensin receptor 1 (AT1R), were pre-treated with increasing concentrations of FR for 30 minutes and subsequently stimulated with 10nM of angiotensin II (AT II) for 2 minutes before BRET response was measured. (**c**) HEK293 cells were pre-treated with increasing concentrations of FR for 1 h, before cells were stimulated with 100 µM carbachol for 30 minutes and intracellular IP1 production was quantified. (**d,e**) HEK293 cells transiently co-transfected with pcDNA for the ORL1 receptor and (**d**) Gα$_{14}$ or (**e**) Gα$_{16}$ were pre-treated with different concentrations of FR for 1 h prior to stimulation with nociceptin for 30 minutes, and intracellular IP1 production was quantified. Shown are mean values ± SEM of 3-7 independent experiments.

## Supplementary Figure 7

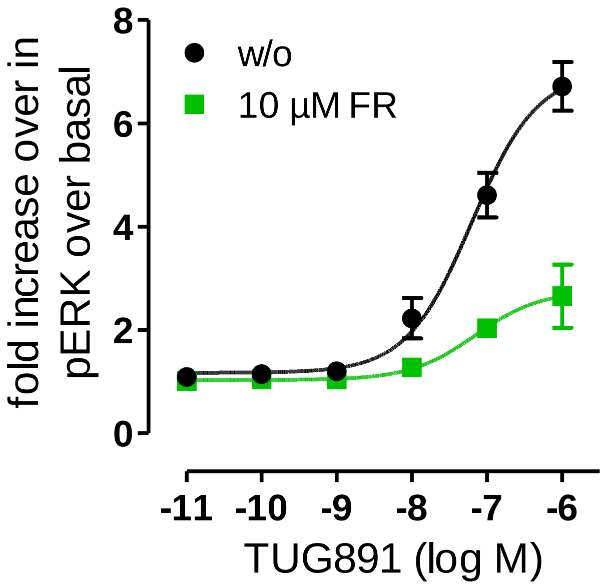


**Supplementary Figure 7: Activation of ERK1/2 phosphorylation in FFA4-CHO-Flp-In™ cells is compromised by 10 µM FR.** pERK (phospho-ERK) was quantified in cells treated with FFA4 agonist TUG891 [1] in the absence and presence of 10 µM FR. Shown are mean values ± SEM of 2 independent experiments, each performed in duplicate. w/o, without FR.

# Supplementary Figure 8

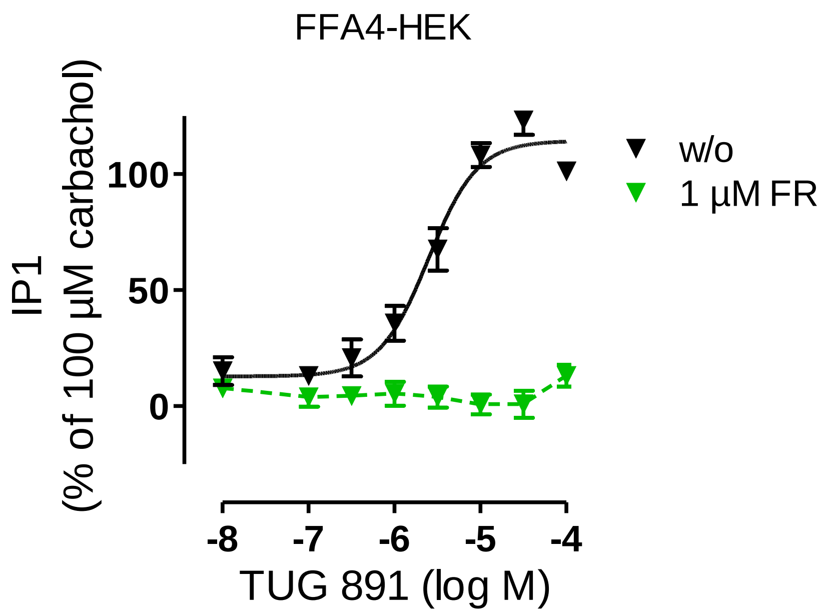


**Supplementary Figure 8: FFA4-mediated inositolphosphate (IP1) production is entirely blocked by FR.** FFA4-HEK cells were pre-treated for 1 h with 1 µM FR and IP1 accumulation was quantified in response to small molecule FFA4 agonist TUG891[1]. Data shown are means ± SEM of 3 independent experiments, each conducted at least in duplicate. w/o, without FR.

# Supplementary Figure 9

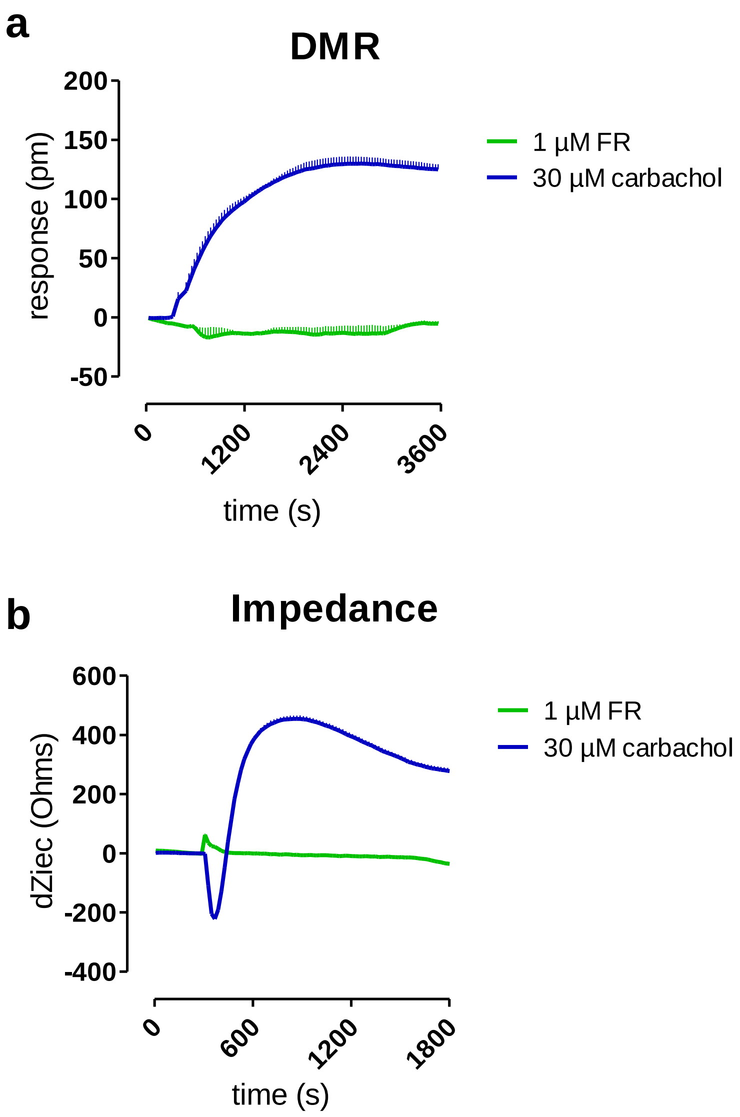


**Supplementary Figure 9: Gq inhibitor FR does not elicit detectable cell activation on its own in label-free holistic cell recordings.** HEK293 cells were treated with 1 µM FR and (**a**) DMR or (**b**) bioimpedance was recorded over time. Carbachol acting via endogenously expressed muscarinic M3 receptors served as viability control. Shown are individual optical response profiles from single experiments (mean values + SEM) that were performed in triplicate and repeated at least twice.

# Supplementary Figure 10

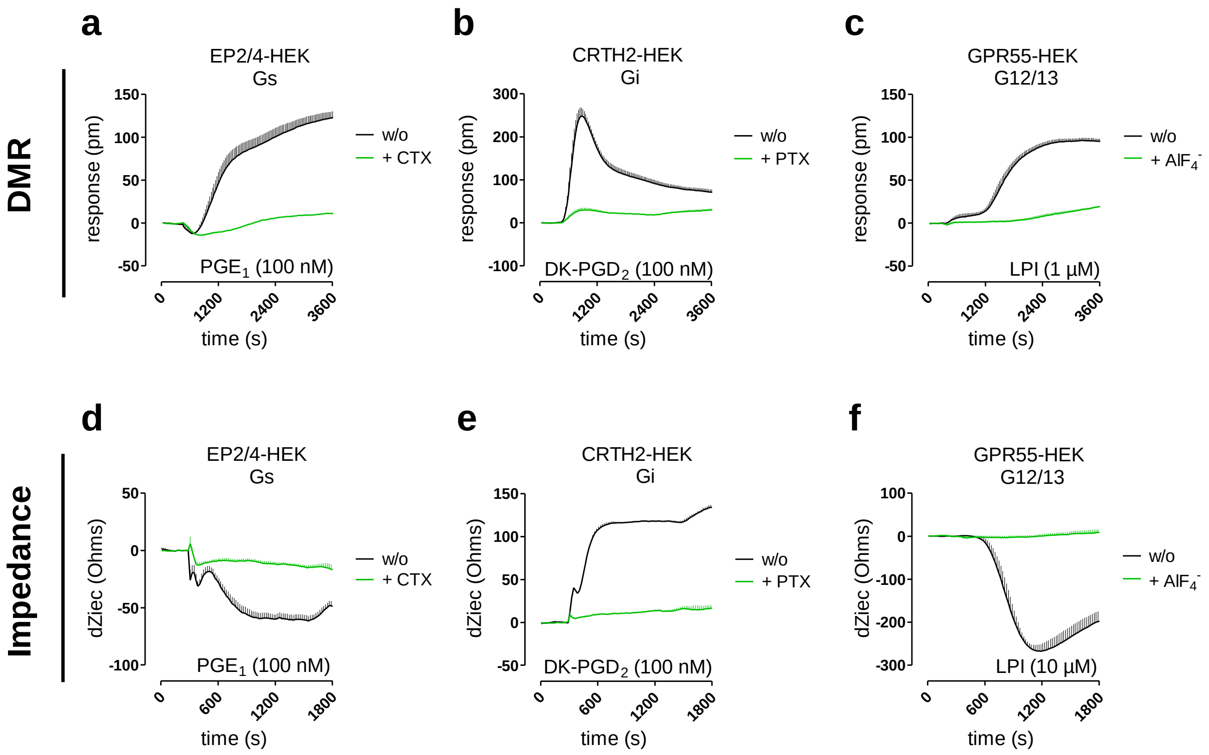


**Supplementary Figure 10: Pharmacological modulation of signaling pathways unveils G protein origin for Gs-, Gi-, and G12/13 stimuli in label-free whole cell sensing.** (**a**,**d**) Pretreatment of cells with choleratoxin (CTX, 200 ng ml$^{-1}$) masks signaling of endogenously expressed Gs-sensitive EP2/4 receptors in response to 100 nM $PGE_1$ in (**a**) DMR or (**d**) impedance assays. (**b**,**e**) Pretreatment with Gi inhibitor PTX (50 ng ml$^{-1}$) silenced signaling of Gi-sensitive CRTH2 upon treatment with agonist DK-$PGD_2$ in CRTH2-HEK cells using (**b**) label-free DMR or (**e**) bioimpedance sensing. (**c**,**f**) Pretreatment of HEK cells stably expressing G12/13-linked GPR55 with pan-G protein activator $AlF_4^-$ (300 µM) are unresponsive to the cognate GPR55 agonist lysophosphatidylinositol (LPI) in (**c**) DMR and (**f**) impedance assays.

Data shown are representative data (means + SEM) of at least three independent experiments, each performed in triplicate, w/o= without pharmacological pathway modulator.

# Supplementary Figure 11

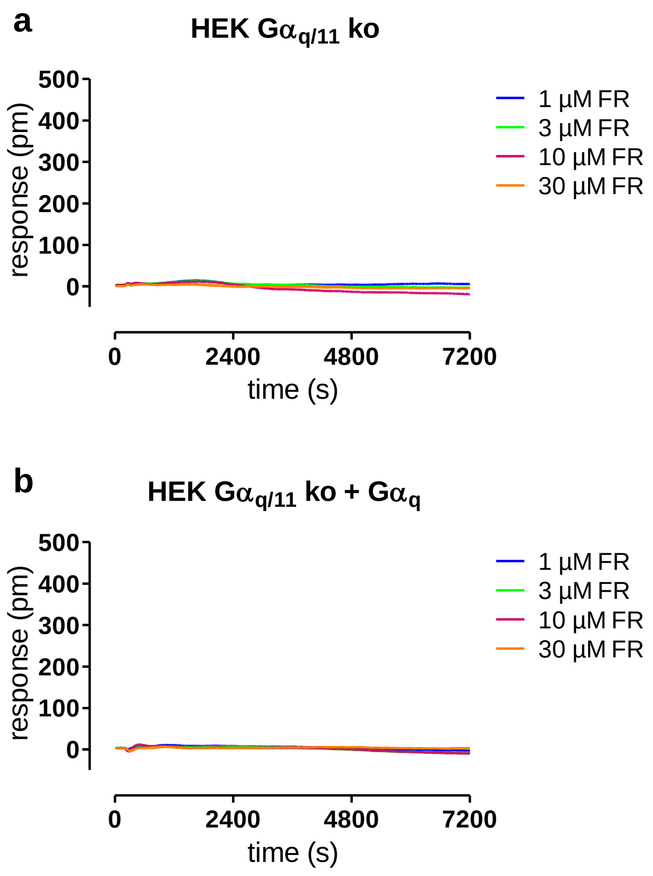


**Supplementary Figure 11: FR does not display any cellular response in HEK cells irrespective of the presence of Gα$_{q/11}$.** Dynamic Mass Redistribution (DMR) induced by supramaximal concentrations of FR in HEK Gα$_{q/11}$ ko cells transfected with either (a) pcDNA3.1(+) or (b) Gα$_q$. Data shown are representative data (means + SEM) of three independent experiments, each performed in triplicate.

# Supplementary Figure 12

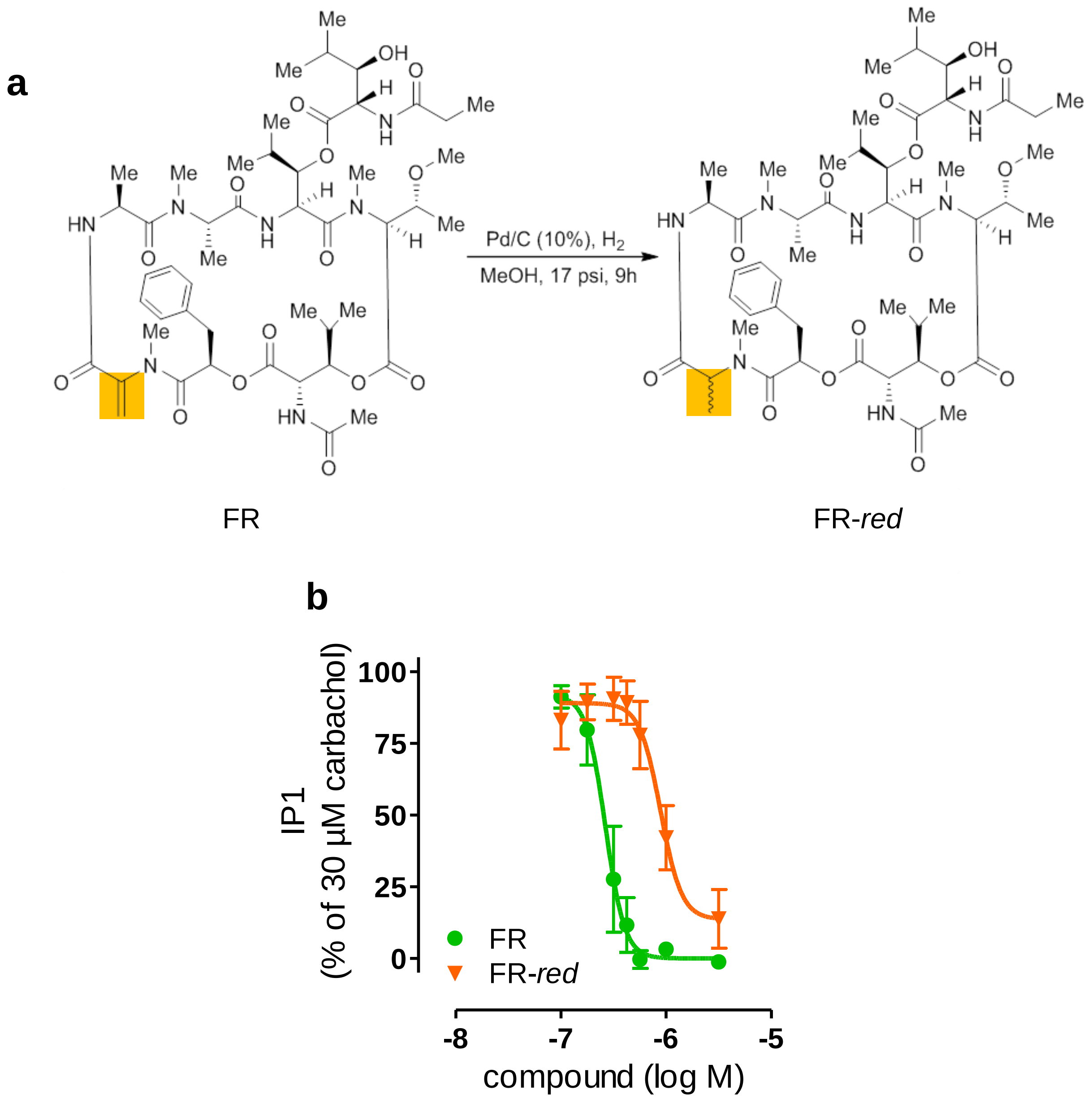


**Supplementary Figure 12: Catalytic hydrogenation of FR to produce "FR-*red*" and determination of its Gq-inhibitory activity.** (**a**) The exocyclic double bond (yellow box) of FR was hydrogenated by hydrogen gas in the presence of palladium/carbon as a catalyst in a pressure tube in analogy to a procedure published for the hydrogenation of YM[2]. The structure of the product FR-*red* was confirmed by high performance liquid chromatography coupled to electrospray ionization mass spectrometry (LCMS) and by $^{1}H$- and $^{13}C$-NMR spectra. (**b**) M1-CHO cells were pre-incubated with increasing concentrations of FR or

FR-*red* and IP1 accumulation was determined in response to carbachol (30 µM). Inhibition of Gq protein activation was slightly less potent when FR-*red* ($pIC_{50}$ 6.05 ± 0.06) was applied instead of FR ($pIC_{50}$ 6.58 ± 0.05). Data are means ± SEM of at least three independent experiments each performed in triplicate.

# Supplementary Figure 13

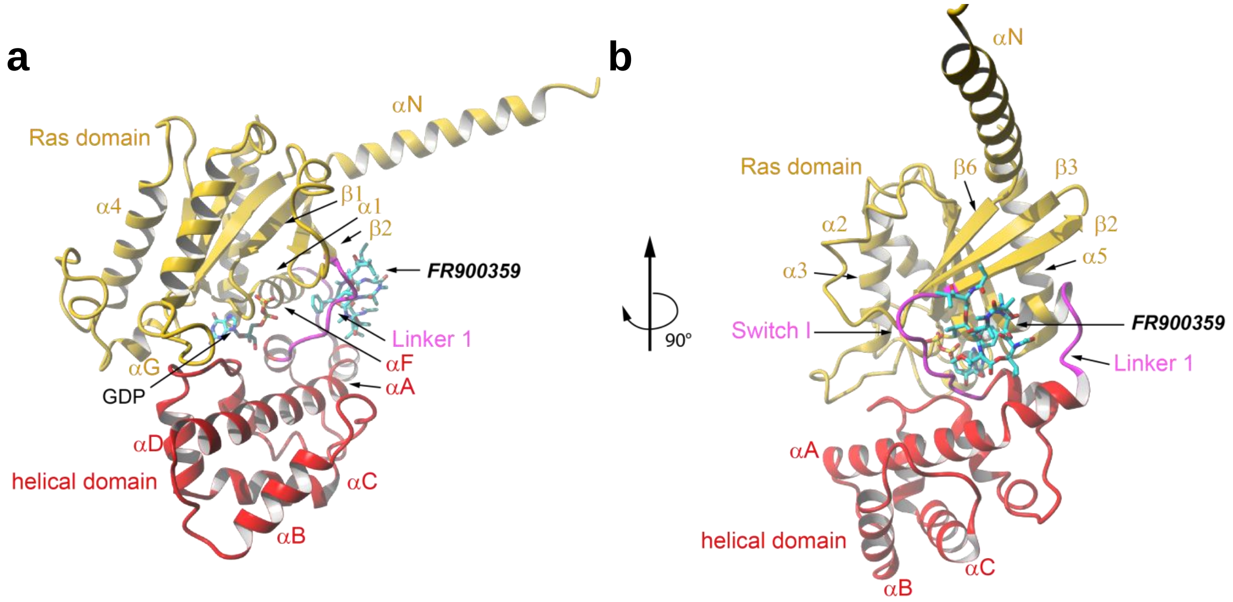


**Supplementary Figure 13**: **FR binding site at Gα$_q$, side view (left) and front view (right**). As revealed by our docking studies, FR900359 binds to the hydrophobic cleft between the helical and Ras domain which is identical with the binding site of YM254890.

# Supplementary Figure 14

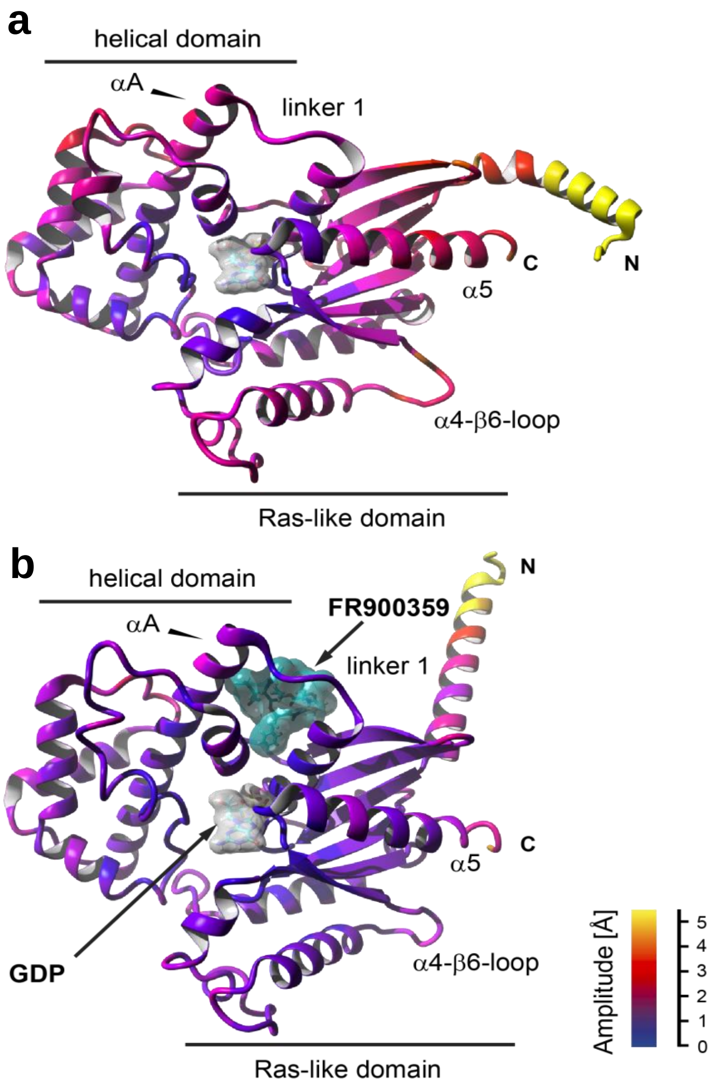


**Supplementary Figure 14**: **Color coded amplitude of flexible protein regions according to the calculated average root mean square fluctuation (RMSF).** Comparison of (**a**) G$\alpha_q$ and (**b**) G$\alpha_q$ bound to FR. GDP and FR are shown by surface representation in light gray and cyan, respectively.

# Supplementary Figure 15

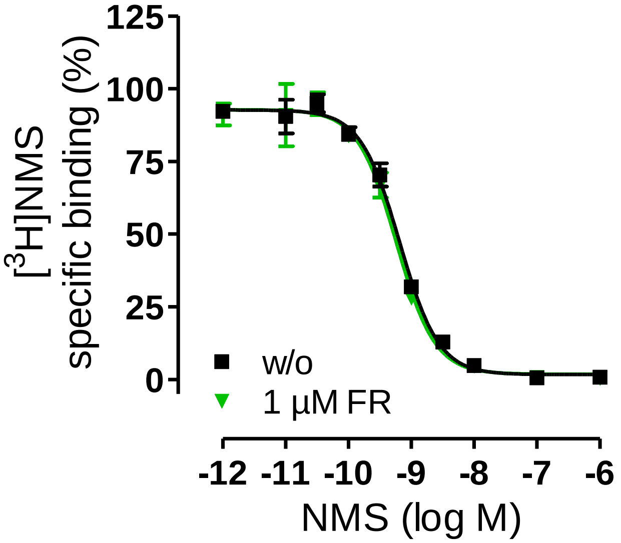


**Supplementary Figure 15: FR does not compromise antagonist recognition of the muscarinic M1 receptor.** Membranes isolated from M1-CHO cells were labeled with 0.2 nM [$^3$H]NMS and homologous competition binding experiments were performed in the absence and presence of 1 µM FR. Shown are mean values ± SEM of at least three independent experiments, performed in duplicate.

# Supplementary Figure 16

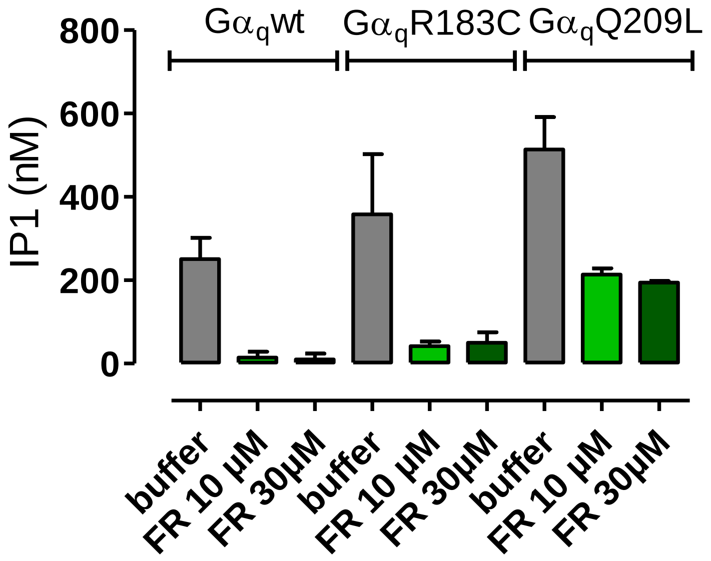


**Supplementary Figure 16: Forced expression of the constitutively active mutants Gα$_q$R183C and Gα$_q$Q209L leads to elevation of intracellular IP1 that is sensitive to FR.** HEK293 cells were transiently transfected with pcDNA3.1 vectors coding for either Gα$_q$wild-type (6 µg 21 cm$^{-2}$ dish), Gα$_q$R183C (1.5 µg 21 cm$^{-2}$ dish), or Gα$_q$Q209L (0.02 µg 21 cm$^{-2}$ dish). Intracellular IP1 production was measured without (buffer) or after treatment with 10 and 30 µM FR for 2 h. Total intracellular IP1 content is given in nM per 30,000 cells. Data shown are mean values + SEM of at least two experiments, each performed in triplicate.

# Supplementary Figure 17

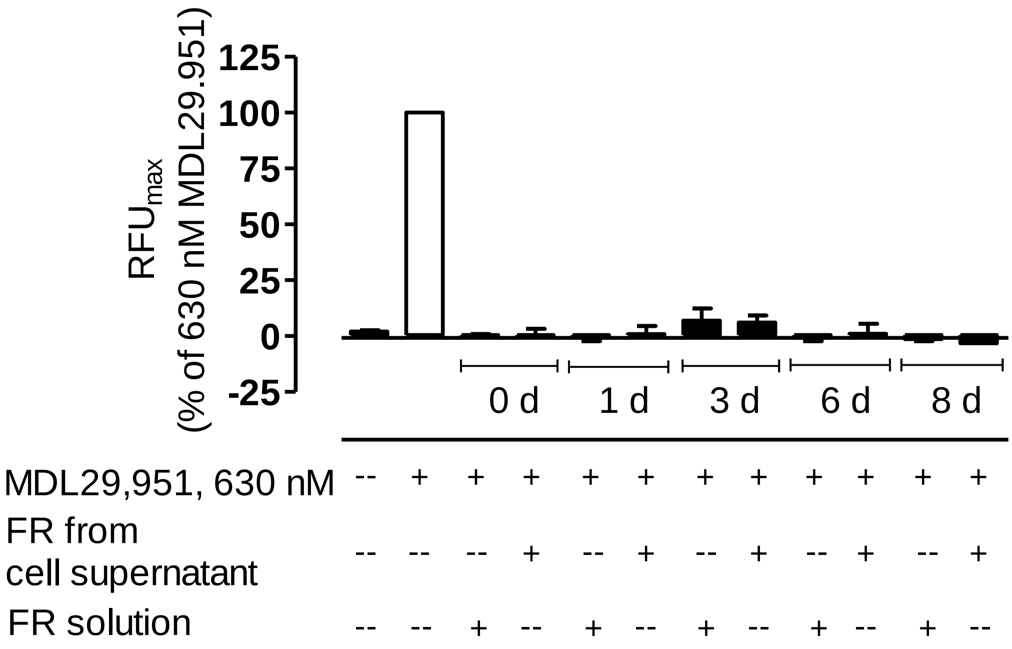


**Supplementary Figure 17: Gq-mediated $Ca^{2+}$ mobilization verifies FR activity over a period of up to eight days.** HEK cells stably expressing a rat ortholog of GPR17 (rGPR17-HEK cells), a $Ca^{2+}$ mobilizing receptor, were stimulated with the small molecule agonist MDL29,951[3] in the absence and presence of 1 μM FR maintained in either Hank's buffered salt solution (HBSS) + 20 mM HEPES "FR-solution" or cell culture supernatant for the indicated time period at 37°C. Data shown are means + SEM of two independent experiments, each performed in triplicate. d= days.

# Supplementary Figure 18

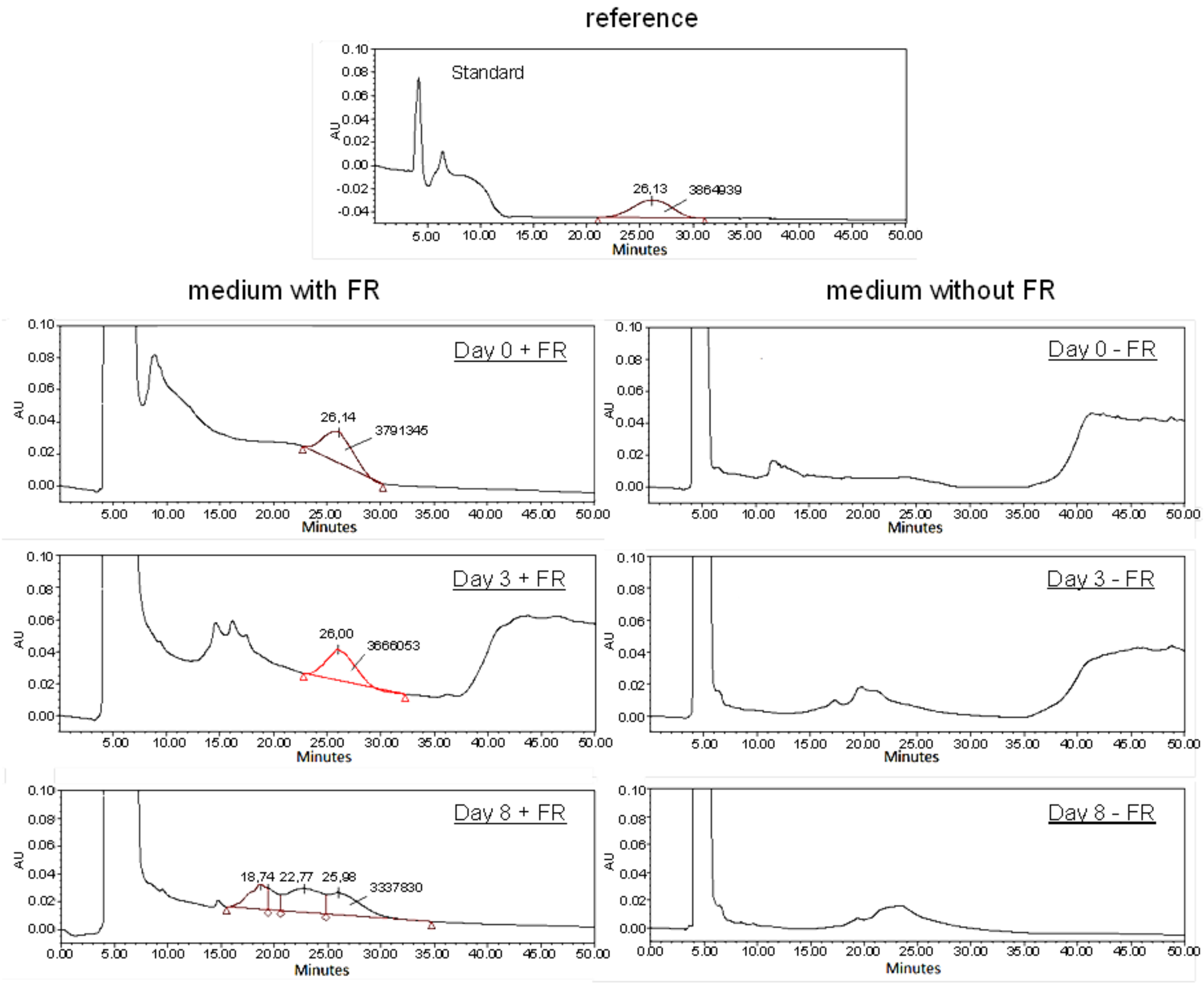


| Sample (c = 100 µM) | Retention Time of FR | Area (AU) of FR |
|---|---|---|
| **Standard FR** | 26.13 min | 3864939 |
| **Day 0 Medium + FR** | 26.14 min | 3791345 |
| **Day 0 Medium - FR** | - | - |
| **Day 3 Medium + FR** | 26.00 min | 3666053 |
| **Day 3 Medium - FR** | - | - |
| **Day 8 Medium + FR** | 25.98 min | 3337830 |
| **Day 8 Medium - FR** | - | - |

**Supplementary Figure 18: HPLC analyses of the stability of FR in cell culture medium.** Concentration of FR in Dulbecco's modified Eagle's medium (DMEM) was analyzed at day zero and after three and eight days by means of an external standard (100 µM FR in methanol). In order to check potential chemical, degradative reactions in the medium over the period of eight days, the medium was also analyzed at day zero, three, and eight without addition of FR. FR is stable in the medium over the period of eight days (a decrease of the integrated area of the FR peak after eight days of around 12% in comparison to day zero can be explained with inaccuracies in integration due to additional peaks in the chromatogram). These additional peaks are derived from chemical reactions of medium components and are not derivatives of FR as indicated by a comparison of the chromatograms of the medium with and without addition of FR.

Data presented are values from one representative experiment.

# Supplementary Figure 19

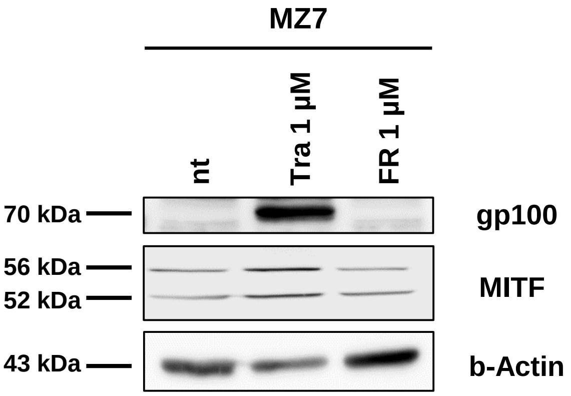


**Supplementary Figure 19: Western blot analysis of the melanoma differentiation markers gp 100 and microphthalmia transcription factor (MITF) in MZ7 cells.** In contrast to the MEK inhibitor Trametinib (Tra), FR did not induce any signs of differentiation in this cell line.

Shown is one representative Western blot.

# Supplementary Figure 20

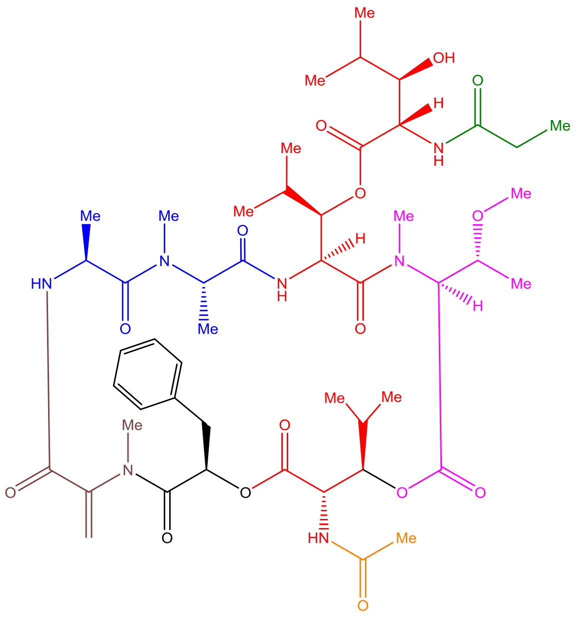

FR900359

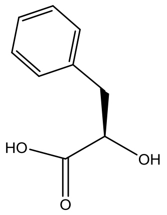

phenyllactic acid

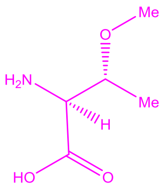

O-methyl-threonine

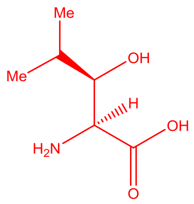

3 x hydroxy-leucine

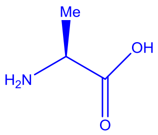

2 x alanine

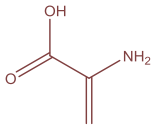

dehydroalanine

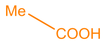

acetic acid

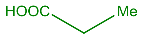

propionic acid

**Supplementary Figure 20: Building blocks of FR.** FR consists of an N-methylated dehydroalanine (grey), an N-methylated O-methyl-threonine (magenta), one unit of phenyllactic acid (black), two alanine moieties (blue), one of which is N-methylated, and three units of hydroxy-leucine (red), one of which is acetylated (slight brown) and the other propionylated (green) at the amino function.

# Supplementary Figure 21

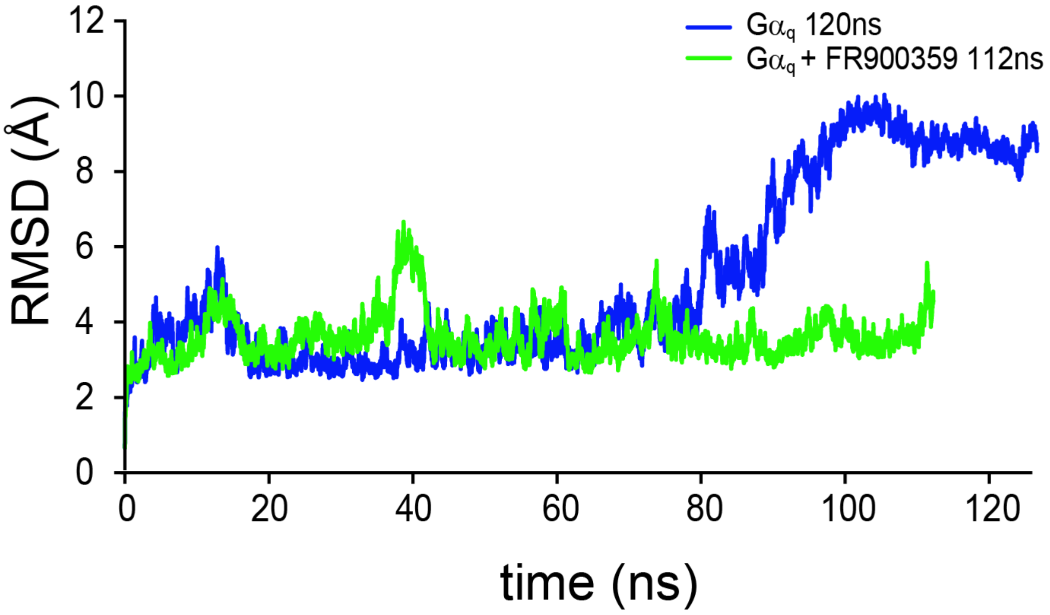


**Supplementary Figure 21: RMSD (heavy atom) time trace of Gα$_q$ and Gα$_q$-FR simulation.** In contrast to the Gα$_q$ simulation without FR (blue line) the RMSD time trace of the Gα$_q$-FR simulation represents almost no structural changes with respect to the starting structure over the time course of about 112 ns indicating a strong reduction of Gα$_q$ domain flexibility.

# Supplementary Table 1

| time (min) | $logEC_{50}$ | SEM |
|---|---|---|
| 30 | -7.81 | 0.43 |
| 60 | -7.91 | 0.07 |
| 90 | -7.88 | 0.14 |
| 120 | -7.81 | 0.05 |

**Supplementary Table 1: $logEC_{50}$ values of the inhibitory effect of FR on [$^{3}$H]GDP dissociation were independent of the time point used for quantification.**

[$^{3}$H]GDP binding to purified $G\alpha_q$ proteins was quantified in presence of increasing concentrations of FR after four different time points (30 min, 60 min, 90 min, 120 min). Data were analyzed by fitting [$^{3}$H]GDP binding *versus* FR concentration to the four parameter logistic function and the logarithm of the half maximal effective concentrations ($logEC_{50}$ expressed as means ± SEM from the respective curves in Fig. 7h) for FR-induced inhibition of [$^{3}$H]GDP dissociation are presented.

# Supplementary Table 2

| | B-RafV600E | N-RasQ61K | G$\alpha_q$R183C | G$\alpha_q$Q209L | G$\alpha_{11}$R183C | G$\alpha_{11}$Q209L |
|---|---|---|---|---|---|---|
| **MZ7** | mutated | --- | --- | --- | mutated | --- |
| **Skmel28** | mutated | --- | --- | --- | --- | --- |
| **Mamel65** | --- | mutated | --- | --- | --- | --- |
| **Mamel119** | --- | --- | --- | --- | --- | --- |
| **Mamel15** | --- | --- | --- | --- | --- | --- |
| **Hcmel12** | --- | --- | --- | --- | --- | mutated |
| **B16** | --- | --- | --- | --- | --- | --- |

**Supplementary Table 2: Mutational status of the seven melanoma cells applied in this study regarding mutant G$\alpha_{q/11}$ alleles and key MAP kinase pathway drivers B-Raf and N-Ras.**

**--- wild type for this allele**

# Supplementary References


1. Hudson, B. D. *et al.* The pharmacology of TUG-891, a potent and selective agonist of the free fatty acid receptor 4 (FFA4/GPR120), demonstrates both potential opportunity and possible challenges to therapeutic agonism, *Mol. Pharmacol.* **84,** 710–725 (2013).
2. Taniguchi, M. *et al.* YM-254890 analogues, novel cyclic depsipeptides with Galpha(q/11) inhibitory activity from Chromobacterium sp. QS3666, *Bioorg. Med. Chem.* **12,** 3125–3133 (2004).
3. Hennen, S. *et al.* Decoding signaling and function of the orphan G protein-coupled receptor GPR17 with a small-molecule agonist, *Sci. Signal.* **6,** ra93 (2013).
